# A meta-ethnography to understand the experience of living with urinary incontinence: ‘is it just part and parcel of life?’

**DOI:** 10.1186/s12894-019-0555-4

**Published:** 2020-01-16

**Authors:** Francine Toye, Karen L. Barker

**Affiliations:** 1 Nuffield Orthopaedic Centre, Oxford University Hospitals NHS Foundation Trust, Windmill Road, Oxford, OX3 7HE UK; 2 Nuffield Department of Orthopaedics, Rheumatology and Musculoskeletal Sciences (NDORMS), University of Oxford, Oxford, UK

**Keywords:** Incontinence, Qualitative research, Qualitative evidence synthesis, Meta-ethnography

## Abstract

**Background:**

Urinary incontinence (UI) is highly prevalent and affects the lives of many men and women. We aimed to conduct a qualitative evidence synthesis (QES) to explore the experience of living with UI and to develop a conceptual model that can help us to understand this experience, and the potential barriers to appropriate healthcare.

**Methods:**

We used the methods of meta-ethnography developed by Noblit and Hare and recently refined for larger studies. Meta-ethnography involves identifying concepts from the studies and abstracting these concepts into a line of argument. We searched for studies that explored the experience of adults with UI. We used the GRADE-CERQual framework to assess confidence in review findings.

**Results:**

We screened 2307 titles, 429 abstracts, 107 full texts and included 41 studies (36 unique samples) in the synthesis. We organised the concepts into 26 conceptual categories, which we further abstracted into 6 themes: (1) Am I ill or is this normal? (2) It effects who I am and how I feel; (3) I feel stigmatised, ashamed and guilty; (4) talking can be difficult but it can help; (5) keeping incontinence under control; (6) have I got to the point that I need help? Our model conceptualises living with UI as navigating antagonists: Is UI normal or am I ill? Do I need help or am I managing? Do I keep UI to myself (and manage alone) or do I tell other people (and get the support that I need)? Do I use control strategies that focus on concealing (avoid risky situations, wear pads) versus, I use strategies that focus on improving the bodily function to improve continence. Our model highlights the experience of stigma, shame and guilt which exert a pull towards concealment.

**Conclusions:**

The culture of secrecy and profound sense of shame is barrier to seeking help. An environment which reduces the shame and stigma of UI may help people to switch the focus to strategies that will improve continence, rather than conceal incontinence.

## Background

Urinary incontinence (UI) is defined by the International Continence Society as ‘the complaint of any involuntary leakage of urine’. UI is highly prevalent and experienced by approximately one quarter of women [1] and potentially half as many men [2]. UI is a very broad term and may include stress, urgency, and mixed UI as well as UI of other types; for example, UI with a neurological diagnosis. Interventions for UI are chosen based on diagnosis and adults (in the absence of a neurological diagnosis) may be offered some type of voiding programme, frequency and urgency strategies, in combination with pelvic floor muscle training (PFMT). However, even though interventions such as PFMT can improve UI [3] it may be that only a minority of people with UI seek help [4]. Those that seek help, they will tend to consult a primary health care clinician, and some will never be referred to a clinician with training in continence management.

The Cochrane Qualitative Research Methods Group acknowledges the importance of including qualitative findings within evidence based healthcare [5]. Qualitative evidence synthesis (QES) can bring together qualitative research findings to make them accessible for public, policy, practice and education. We wanted to explore people’s experience of living with UI in order to further understand its impact on the lives of men and women, and to understand some of the barriers to seeking help. We aimed to conduct a qualitative evidence synthesis (QES) and to develop a conceptual model that can help us to understand some of the barriers to appropriate healthcare [6]. There are likely to be differences in the experience of UI for men and women of different ages with diverse pathophysiology, we wanted to explore themes that cut across this experience and to identify areas for further research.

## Methods

We used the 7 stages of Meta-ethnography developed, refined and reported by Toye and colleagues [7, 8] and the recent eMERGe meta-ethnography reporting guidelines as a guide for our report [9].

### Selecting meta-ethnography and getting started (stage 1)

This phase incorporates the rationale and aims of the study. Meta-ethnography, first developed by Noblit and Hare [6] has provided insight into a range of healthcare experiences including chronic musculoskeletal pain [10], rheumatoid arthritis [11], fibromyalgia [12], osteoporosis [13] and chronic pelvic pain [14]. There are various methods for QES [15–19]. Some focus on amalgamating and describing findings: other, such as meta-ethnography, aim to develop conceptual understandings through a process of constant comparison and abstraction [6]. We planned to develop a line of argument synthesis, and thus make ‘a whole into something more than the parts alone imply’ [6] (page 28). We first identified any existing QES, using search terms developed for this purpose [20, 21]. We identified three QES that explored specific areas of UI: French and colleagues explored the uptake and delivery of behavioural interventions for UI (6 studies) [22]; Hay-Smith and colleagues explore the adherence to PFMT (13 studies) [23]; Avery and colleagues explored the psychosocial aspects of living with UI (10 studies) [24]. We also found two QES that explored the global experience of UI: Siddiqui and colleagues explored ethnic and racial differences [25] (*n* = 23 studies); Mendes and colleagues explored women’s experience of living with UI (*n* = 30 studies) using the Joanna Briggs Institute Qualitative Appraisal and Review Instrument (JBI-QARI) [26]. JB-IQARI is a descriptive method for QES. The innovation of our study is: (1) to undertake a conceptual synthesis using the methods of meta-ethnography; (2) to incorporate the experience of men from published qualitative findings.

### Deciding what is relevant (stage 2)

We included primary qualitative studies that explored the experience of men and women with UI. UI is a broad term and we intended to be broad in our inclusion. We used subject headings and free text terms for qualitative research, combined with subject heading and free text terms for incontinence (Table 1). Our search terms were adapted from the InterTASC Information Specialists’ Sub-Group (ISSG) Search Filter Resources [27–30] and have been used in other meta-ethnographies [7, 31, 32] We excluded studies that exclusively explored: peri-partum, neurological, faecal, long-term care, acute hospitalisation, pelvic organ prolapse and incontinence surgery. In their original text, Noblit and Hare do not advocate an exhaustive search [6] and the number of studies included in meta-ethnographies ranges [16, 18, 33]. We took a selective approach to searching in order to be purposeful, yet also comprehensive enough to develop rich ideas. Similarly, some qualitative reviewers suggest a more targeted approach to searching. For example, you could stop searching when collecting additional data adds no more insight (theoretical saturation) [34]. This approach is more comparable with the sampling strategies for qualitative research. One option might be to start your search with a single data base and expand as analysis proceeds [17]. Our previous QES indicate that Medline retrieves the majority of studies [7, 13, 14, 35]. We therefore limited our initial search to one bibliographic database (Medline) from inception to January 2017 and subsequently updated the search to include studies to February 2019. Campbell and colleagues suggest that 38 studies is adequate for meta-ethnography [33] . Our plan was to widen our search if the initial search did not identify this many studies, or if analysis did not yield rich ideas. Table 1 reports the elements of STARLITE (sampling strategy, type of study, approaches, range of years, limits, inclusion and exclusions, terms used, electronic sources) for reporting qualitative literature searches [36].

**Table 1:** Starlite Report

STARLITE CATEGORY	DESCRIPTION
Sampling Strategy	Selective
Type of studies	Qualitative research, fully reported
Approaches	Single electronic medical database (medline)
Range of years	1990 to January 2017; updated Feb 2019
Limits	[Human age groups Young adult OR Adult OR Middle aged OR Aged Or Aged, 80 and over] [Languages English]
Inclusion and exclusions	Incontinence Excluded: peri-partum, neurological, faecal, long-term care and acute hospitalisation, pelvic organ prolapse, incontinence surgery
Terms used	((exp “FOCUS GROUPS”/ OR exp. “ANTHROPOLOGY, CULTURAL”/ OR exp. “QUALITATIVE RESEARCH”/ OR exp. “INTERVIEWS AS TOPIC”/ OR exp. “ATTITUDE TO HEALTH”/ OR exp. “NURSING METHODOLOGY RESEARCH”/ OR (Qualitative ADJ5 (theor* OR study OR studies OR research OR analysis)).ti,ab OR (ethno*).ti,ab OR (emic OR etic).ti,ab OR (phenomenolog*).ti,ab OR (hermeneutic*).ti,ab OR (heidegger* OR husserl* OR colaizzi* OR giorgi* OR glaser OR strauss OR (van AND kaam*) OR (van AND manen) OR ricoeur OR spiegelberg* OR merleau).ti,ab OR (constant ADJ3 compar*).ti,ab OR (focus ADJ3 group*).ti,ab OR (grounded ADJ3 (theor* OR study OR studies OR research OR analysis)).ti,ab OR (narrative ADJ3 analysis).ti,ab OR (discourse ADJ3 analysis).ti,ab OR ((lived OR life) ADJ3 experience*).ti,ab OR ((theoretical OR purposive) ADJ3 sampl*).ti,ab OR (field ADJ (note* OR (field ADJ record*) OR fieldnote*)).ti,ab OR (participant* ADJ3 observ*).ti,ab OR (action ADJ research).ti,ab OR ((co AND operative) AND inquir*).ti,ab OR (co-operative AND inquir*).ti,ab OR (cooperative AND inquir*).ti,ab OR ((semi-structured OR semistructured OR unstructured OR structured) ADJ3 interview*).ti,ab OR ((informal OR in-depth OR indepth OR “in depth”) ADJ3 interview*).ti,ab OR ((“face-to-face” OR “face to face”) ADJ3 interview*).ti,ab OR (“IPA” OR “interpretive phenomenological analysis”).ti,ab OR (“appreciative inquiry”).ti,ab OR (social AND construct*).ti,ab OR (poststructural* OR post structural* OR post-structural*).ti,ab OR (postmodern* OR post modern* OR post-modern*).ti,ab OR (feminis*).ti,ab OR (humanistic OR existential OR experiential).ti,ab) AND (exp ENURESIS/ OR exp. ENCOPRESIS/ OR exp. “FECAL INCONTINENCE”/ OR exp. “ABSORBENT PADS”/ OR exp. “URINARY INCONTINENCE”/ OR exp. “NOCTURNAL ENURESIS”/ OR exp. “DIURNAL ENURESIS”/ OR exp. “URINARY INCONTINENCE, STRESS”/ OR exp. “URINARY INCONTINENCE, URGE”/ OR exp. “URINARY BLADDER, OVERACTIVE”/ OR (incontinence).ti,ab OR (enuresis).ti,ab OR (encopresis).ti,ab OR ((overactiv* OR “over active” OR over-activ*) ADJ5 bladder).ti,ab))
Electronic sources	Medline

A single reviewer screened potential articles for potential relevance. A second researcher did not verify the screening for the following reasons: (1) qualitative research methods do not hinge upon statistical analysis of an entire data set; (2) conceptual reviews such as mega-ethnography focus on conceptual analysis. We feel that, in reviews of this scale, research time is more productively spent on data extraction and analysis, rather than being exhaustive. (3) The reviewer had the experience to recognise a qualitative study. To determine if studies were *good enough* to be included in analysis, both reviewers appraised studies, as suggested by Dixon-Woods, as: ‘irrelevant’, ‘fatally flawed’, ‘satisfactory’, or ‘key’ (conceptually rich) [37]. We excluded ‘irrelevant’ and ‘fatally flawed’ studies. Fatally flawed studies included those that had very limited qualitative data. Although appraisal tools are often used in qualitative synthesis [18], QES reviewers do not always use them to determine inclusion [33] and there tends to be low agreement between reviewers [37]. Our approach to quality appraisal for QES is described fully in Toye and colleagues [38].

### Reading included studies (stage 3)

We used NVivo 11 software for qualitative analysis to facilitate close reading of the studies and to keep track of the analytical decisions [39]. Using NVivo allows you to link originating text with your findings. We extracted contextual information about each study (location, condition, age, gender, study aim, recruitment and methods) to determine how studies were related. Once we had decided which studies to include, two reviewers closely read the full studies in alphabetical order by author, to identify the *ideas* or concepts from each primary study. Close reading is an approach which challenges the reviewer to critically appraise the intentions of the author, and ask what does this mean and what it is an example of? Schütz [33] distinguishes between (1) *first-order constructs* (the participants’ ‘common sense’ interpretations in their own words) and (2) *second order constructs* (the researchers’ interpretations of first order constructs). In meta-ethnography, the ‘data’ are second order constructs. These are the ideas or concepts that are further abstracted to develop *third order constructs* (reviewer’s interpretations of second order constructs) which are the QES findings. We excluded data from analysis if both reviewers could not decipher a clear concept. We then rewrote the concepts in the first person. Writing in the first person facilitates the use of accessible language, and we have found it a powerful way for readers (and reviewers) to fully engage in the meaning and sentiment of the ideas.

### Determining how studies are related and translating studies (stages 4 and 5)

Once we had a list of second order concepts, two reviewers ‘translated’ [6] these by comparing similarities and differences and gradually organising them into *conceptual categories*. The aim of constant comparison is to distil ‘the essence of an idea’ [20]. This process of abstraction is integral to qualitative analysis [40]. Other meta-ethnographies [33], researchers have used themes from an ‘index’ paper as a template to organise the analysis [41]. We did not use an index paper as there are also issues about how to decide which paper is *index* [38], and also this can be cumbersome when translating a large number of studies. It is not uncommon to find that some concepts from primary studies do not fit or add anything to a conceptual analysis and, for transparency, these are reported. When both reviewers had agreed on a description of each conceptual category, one reviewer rewrote the descriptions in the first person and printed them on cards. Both reviewers then organised the cards collaboratively through comparison and discussion to develop the QES findings.

### Synthesising translations and expressing the synthesis (stage 6 and 7)

The final stage of meta-ethnography involves organising final themes into a storyline or conceptual model. We intended to produce a line of argument by developing ‘a grounded theory that puts the similarities and differences between studies into interpretive order’ [6] (page 64). A line of argument can incorporate *similar* and *contrasting* ideas from primary studies, thus allowing both reciprocal *and* refutational translation simultaneously, rather than choosing one or the other. We applied the GRADE-CERQual framework [42–49] to define our confidence in the themes entering the conceptual model as the framework was not designed to be applied to conceptual models. GRADE-CERQual suggest four domains: (1) ‘*Methodological limitations’*; (2) ‘*Relevance’;* (3) ‘*Adequacy of data’* (the ‘degree of richness and quantity of data supporting a review finding’); *(4)* ‘*Coherence’* (consistency across primary studies); and finally, an overall rating of confidence (high, moderate, low, very low).

## Results

**Fig. 1:**
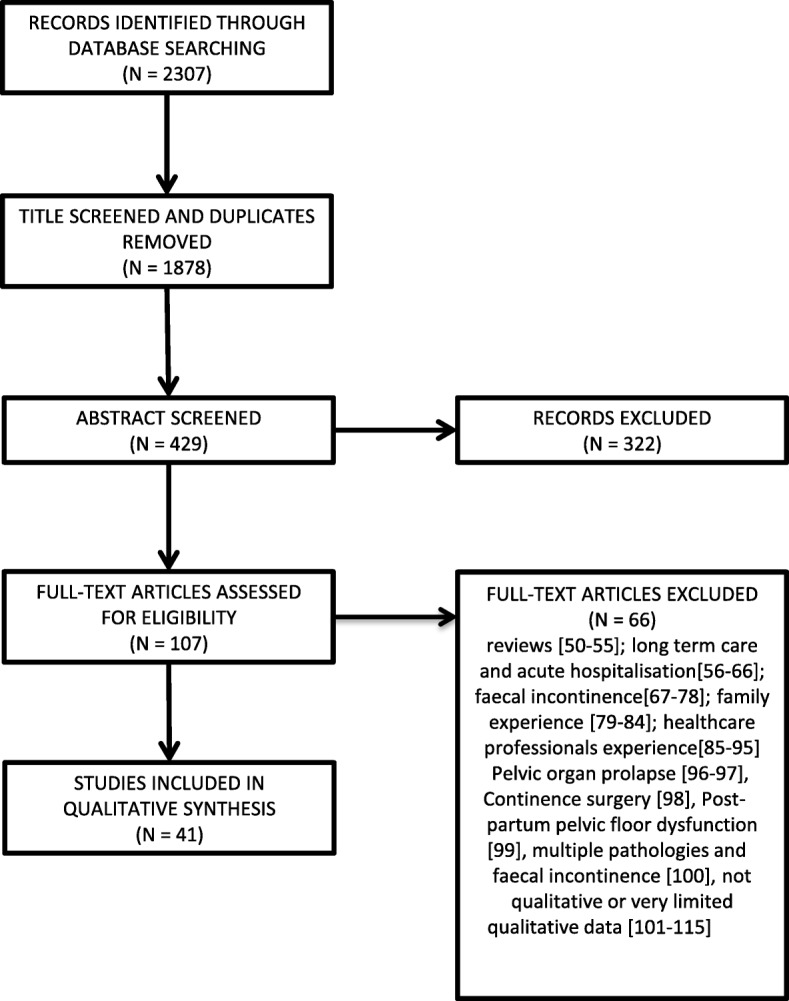
Search findings, This flowchart show the records identified through database searching, those excluded following screening and those included

Figure 1 illustrates the results of our systematic search. We screened 2307 titles, 429 abstracts and 107 full texts. We excluded 66 out of 107: reviews [50–55]; long term care and acute hospitalisation [56–66]; faecal incontinence [67–78]; family experience [79–84]; healthcare professionals’ experience [85–95] Pelvic organ prolapse [96, 97], Continence surgery [98], Post-partum pelvic floor dysfunction [99], multiple pathologies [100], not qualitative or very limited qualitative data [101–115]. We included 41 studies in the final analysis [116–156]. Our purposive search using a single database identified 41 studies in total, including 18 additional studies to Mendes and colleagues [26] and Siddiqui and colleagues [25], who identified 30 and 23 studies respectively. Siddiqui identified an additional 3 studies [157–159] and Mendes identified an additional 7 studies [110, 158, 160–164]. Table 2 shows the overlaps and additional studies found in all 3 QES. Our updated search (February 2019 identified two additional studies [168, 169].

**Table 2:** studies included in QES, indicating overlap in studies identified in other QES on UI

Author, Year	Toye & barker	Mendes & Colleagues [26]	Siddiqui & Colleagues [25]
Anger & Colleagues [107]	Excluded full text		Y
Bradway & Strumpf 2008 [109]	Excluded full text		Y
Bradway 2005 [111]	Excluded full text		Y
Bradway & Colleagues [103]	Excluded full text		Y
Brown & Colleagues 1998 [165]	Excluded on abstract		Y
Chaliha & Stanton 1999 [105]	Excluded full text		Y
Hatchett & Colleagues [157]	Not found		Y
Klemm & Creason 1991 [166]	Excluded on abstract		Y
Welch & Colleagues [158]	Not found		Y
Wells and Wagg 2007 [159]	Not found		Y
Bradway & Barg [110]	Not found	Y	Y
Elstad & Colleagues 2010 [164]	Not found	Y	Y
Akyuz & Colleagues 2014 [160]	Not found	Y	
Coyne & Colleagues 2007 [161]	Not found	Y	
Delarmelindo 2013 [162]	Not found	Y	
Higa & Colleagues [167]	Excluded on abstract	Y	
Macdonald & Butler [59]	Excluded full text	Y	
Roe & May [100]	Excluded full text	Y	
Siu & Lopez [163]	Not found	Y	
Welch & Colleagues [158]	Not found	Y	
Andersson et al. 2008 [116]	Y	Y	Y
Andersson et al. 2009 [117]	Y	Y	Y
Ashworth & Hagan 1993 [118]	Y	Y	
Cochran 1998 [119]	Y		
Doshani et al. 2007 [120]	Y	Y	Y
Dowd 1991 [121]	Y	Y	
Getliffe et al. 2007 [122]	Y		
Gjerde et al. 2013 [123]	Y	Y	
Griffiths et al. 2009 [124]	Y		
Hägglund & Wadensten 2007 [125]	Y	Y	
Hägglund & Ahlström 2007 [126]	Y	Y	
Hamid et al. 2015 [127]	Y	Y	
Hayder & Schnepp 2010 [128]	Y	Y	Y
Hayder 2012 [129]	Y		
Higa et al. 2011 [130]	Y	Y	
Horrocks et al. 2004 [131]	Y		Y
Jackson et al. 2012 [132]	Y		
Kao et al. 2015 [133]	Y		
Komorowski & Chen 2006 [134]	Y	Y	Y
Li, Low & Lee 2007 [135]	Y		Y
Macinnes 2008 [136]	Y		
Mason et al. b2001 [138]	Y		
Mason et al. 1999 [137]	Y	Y	
Milne 2006 [139]	Y		
Nicolson et al. 2008 [140]	Y	Y	
Peake & Manderson 2003 [141]	Y	Y	Y
Peake, Manderson & Potts 1999 [142]	Y		
Roos et al. 2014 [143]	Y	Y	
Sange et al. 2008 [144]	Y	Y	Y
Shaw, William & Assassa 2000 [147]	Y		
Shaw et al. 2001 [145]	Y		
Shaw et al. 2008 [146]	Y		
Siu 2014 [148]	Y		
Siu 2015 [149]	Y		
Skoner & Haylor 1993 [150]	Y	Y	
St John, James & Mckenzie 2002 [151]	Y		
van Den Muijsenbergh & Lagro-Janssen 2006 [152]	Y	Y	Y
Welch, Taubenberger & Tennstedt 2011 [154]	Y		
Welch et al. 2012 [153]	Y		
Wilkinson 2001 [155]	Y		Y
Zeznock, Gilje & Bradway 2009 [156]	Y	Y	

Table 3 shows the country, condition and age of participants, number or participants (number of men), aim of the study and the data collection and methodology of included studies. These studies explore the experience of 1046 participants from a range of countries: UK (9 studies), Australia (8 studies), USA (7 studies), Sweden (4 studies), Germany (2 studies), Hong Kong (3 studies), The Netherlands (2 studies), Brazil (1 study), Canada (1 study), China (1 study), Ethiopia (1 study), Iran (1 study) and Taiwan (1 study). All reports explored women’s’ experience. Eleven reports (9 unique studies) explored the experience of 190 men [128, 129, 131, 132, 139, 140, 145, 146, 151, 153, 154]. However, the reports did not indicate differences in men’s and women’s experience and we have analysed these findings together.

**Table 3:** Description of studies: Author, year, country, condition, age, number of participants (number of men), aim, data collection, methodology, recruitment context, assessment of relevance and quality

Author & Year	Geography	Condition	Age	Number (male)	Aim to explore:	Data collection, methodology	Recruitment	Relevance	Assessment
1. Andersson et al. 2008 [116]	Sweden	UI	66–89	11	Experience of UI among women who do not desire further treatment	Interviews, Phenomenology	District nurse	direct	satisfactory
2. Andersson et al. 2009 [117]	Sweden	UI	30 80+	14	Experience of UI among Syrian women living in Sweden	Focus groups, Phenomenology	Snowball sample	direct	satisfactory
3. Ashworth & Hagan 1993 [118]	UK	UI	25–55	28	Women’s’ experience of UI	Interviews, Phenomenology	newspaper	direct	key
4. Cochran 1998 [119]	USA	UI	60–88	19 (NK)	Experience of UI of older persons living in the community	Interviews, Not stated	Community volunteer/doctor invite	direct	satisfactory
5. Doshani et al. 2007 [120]	UK	UI	36–82	24	Experience of UI among south Asian Indian women in Leicester, UK	Focus groups, Thematic analysis	South Asian community centres	direct	satisfactory
6. Dowd 1991 [121]	USA	UI	58–79	7	Experience of UI and adjustment in older women	Interviews, Grounded theory	‘convenience sample’	direct	satisfactory
7. Getliffe et al. 2007 [122]	UK	UI	29–89	99	Experience of using absorbent products for ‘light’ UI and impact on women’s quality of life	Interviews, Thematic analysis	Incontinence services, consumer organisations and adverts	indirect#	satisfactory
8. Gjerde et al. 2013 [123]	Ethiopia	UI	NK	18^1^	Experience of UI in rural and semi urban settings in Ethiopia	Interviews, Systematic text condensation	part of a Incontinence and Prolapse study.	direct	satisfactory
9. Griffiths et al. 2009 [124]	UK	UI	30–74	22	Experience of physiotherapy sessions for the management of UI	Interviews, Thematic analysis	Embedded in a trial	direct	satisfactory
10. Hägglund & Wadesten [125]	Sweden	UI	34–52	14	Experience of UI	Interviews, Phenomenology	Cohort study; women who had not sought help	direct	satisfactory
11. Hägglund & Ahlström 2007 [126]	Sweden	UI	37–52	13	Experience of UI and health seeking in long-term UI	Interviews, Phenomenology	Cohort study; women who had sought help for UI	direct	satisfactory
12. Hamid et al. 2015 [127]	Iran	UI	52–68	17	Experience of Muslim community-dwelling postmenopausal women of UI	Interviews, Phenomenology	Community snowball sample	direct	satisfactory
13. Hayder & Schnepp ^a^ 2010 [128]	Germany	UI	38–83	32 (10)	Experience of UI in daily life	Interviews, Grounded theory	Community advert	direct	key
14. Hayder^a^ 2012 [129]	Germany	UI	38–83	32 (10)	Experience of UI and impact on sexuality and intimate relationships	Interviews,	Community advert	direct	satisfactory
15. Higa et al. 2011 [130]	Brazil	UI	30–45	8	the meanings of silence for Brazilian women with UI	Interviews, Content analysis,	Community snowball sample	direct	satisfactory
16. Horrocks et al. 2004 [131]	UK	UI	66–94	20 (9)	Why older people living in the community do not seek help with UI	Interviews, Grounded theory	Community survey	partial*	satisfactory
17. Jackson et al. 2012 [132]	USA	UI	NK	144 (71)	How talking with others influences symptom management	Interviews, Thematic analysis	Community survey	direct	satisfactory
18. Kao et al. 2015 [133]	Taiwan	UI	44–66	12	Experiences of PFMT for UI and the impact on their sexuality	Interviews, Thematic analysis	Women who had gone to a PFMT education programme	indirect	satisfactory
19. Komorowski & Chen 2006 [134]	China	UI	24–81	15	Experiences of Chinese women living with UI	Interviews, IPA	Reported UK at Obstetrics and gynaecology department	direct	satisfactory
20. Li, Low & Lee 2007 [135]	Hong Kong	UI	42–77	9	Community-dwelling women’s experiences in coping with UI	Interviews Content analysis	Continence clinic (stress incontinence)	direct	satisfactory
21. Macinnes 2008 [136]	UK	UI	28–65	12	To explore why some women with UI drop out of healthcare	Telephone interviews, Thematic analysis	Continence clinic (stress incontinence)	direct	satisfactory
22. Mason et al.^b^ 1999 [137]	Australia	UI	21–45	52	The effects of stress incontinence on women in their childbearing years	Interviews, Thematic analysis	One year post-partum with UI	partial	satisfactory
23. Mason et al. ^b^2001 [138]	Australia	UI	21–45	52	are women made aware of UI at the time of childbirth and why some do not seek help	Interviews, Thematic analysis	One year post-partum with UI	partial	satisfactory
24. Milne 2006 [139]	Canada	UI	24–86	38 (5)	Self-care strategies in UI and factors that influence their self-care choices	15 interviews/3 focus groups description	Adverts in health clinics, newspapers, health education sessions, clinics	direct	satisfactory
25. Nicolson et al. 2008 [140]	UK	OAB	51–85	18 (8)	Experiences of overactive bladder symptoms	Interviews/focus groups, Thematic analysis	Primary care, adverts	direct	satisfactory
26. Peake & Manderson^c^ 2003 [141]	Australia	UI	40–60	75	Social aspects of UI in women in their middle years.	Interviews, Thematic analysis	Primary care	direct	key
27. Peake, Manderson & Potts^c^ 1999 [142]	Australia	UI	40–60	75	Women’s discourse regarding theirown UI	Interviews, Thematic analysis	Primary care	direct	key
28. Roos et al. 2014 [143]	Netherlands	POP/UI	31–64	37	Impact of pelvic organ prolapse and/or UI on sexual dysfunction	Interviews, Thematic analysis	Scheduled for corrective surgery	indirect	satisfactory
29. Sange et al. 2008 [144]	UK	UI	21–70	9	Religious/cultural influences on help-seeking in south Asian Muslim women	Focus groups, Framework analysis	Language classes	direct	satisfactory
30. Shaw et al. 2001 [145]	Australia	UI	40–63	31 (8)	Help seeking behaviour in people with UI and barriers to service use	Interviews Thematic analysis	Embedded in a continence service trial	indirect	satisfactory
31. Shaw et al. 2008 [146]	UK	UI	41–89	33 (18)	help-seeking in middle and older aged people with UI	Interviews Grounded theory	Community survey	direct	satisfactory
32. Shaw, William & Assassa 2000 [147]	Australia	UI	40–62	23 (7)	Patients’ views of a new nurse led continence service in a randomized trial	Interviews Thematic analysis	Post course led by continence nurse	direct	satisfactory
33. Siu 2014 [148]^d^	Hong Kong	OAB	21–59	30	Doctor-patient communication (female patients and male urologists)	Interviews Thematic analysis	OAB patient self-help group	direct	satisfactory
34. Siu 2015 [149]^d^	Hong Kong	OAB	21–59	30	Reasons behind doctor shopping behaviour in patients with overactive bladder	Interviews Thematic analysis	OAB patient self-help group	direct	satisfactory
35. Skoner & Haylor 1993 [150]	USA	UI	31–50	8	Perceptions of UI	Interviews Grounded theory	Women’s’ magazines or snowball sample	direct	satisfactory
36. St John, James & Mckenzie 2002 [151]	Australia	UI	40–66	11 (5)	Perspectives of a service for community dwelling people with UI	Interviews Thematic analysis	Community health, continence service, home visits	direct	satisfactory
37. van Den Muijsenbergh & Lagro-Janssen 2006 [152]	Netherlands	UI	45 MEAN	30	The impact of UI on Moroccan and Turkish women and their treatment preferences	Interviews Thematic analysis	Primary care, pelvic floor physio, Moroccan care consultants	direct	satisfactory
38. Welch et al. ^e^ 2012 [153]	USA	LUTS	34–85	90 (49)	qualitative methods for developing patient-reported outcomes	Interviews Thematic analysis	Community survey	indirect	satisfactory
39. Welch, Taubenberger & Tennstedt^e^ 2011 [154]	USA	LUTS	34–85	90 (49)	Treatment seeking for lower urinary tract symptoms	Interviews Thematic analysis	Community survey	indirect	satisfactory
40. Wilkinson 2001 [155]	Australia	UI	40–64	6	Experiences of Pakistani women with UI	Interviews Thematic analysis	Continence service	direct	satisfactory
41. Zeznock, Gilje & Bradway 2009 [156]	Alaska	UI	33–86	17	Experiences of Alaskan women living with UI in rural/urban settings	Interviews Thematic analysis	Urological, women’s health and primary care	direct	satisfactory

*OAB* overactive bladder, *POP* pelvic organ prolapse, *LUTS* lower urinary tract infection, *IPA* interpretative phenomenological analysis

^1^ 5/18 constant UI from obstetric fistula, others had mild to continuous leakage; ^*^2 men had permanent indwelling catheters; ^#^ 16/99 linked to other conditions

^a,b,c,d,e^ themes drawn from a single cohort in these studies

**Fig. 2:**
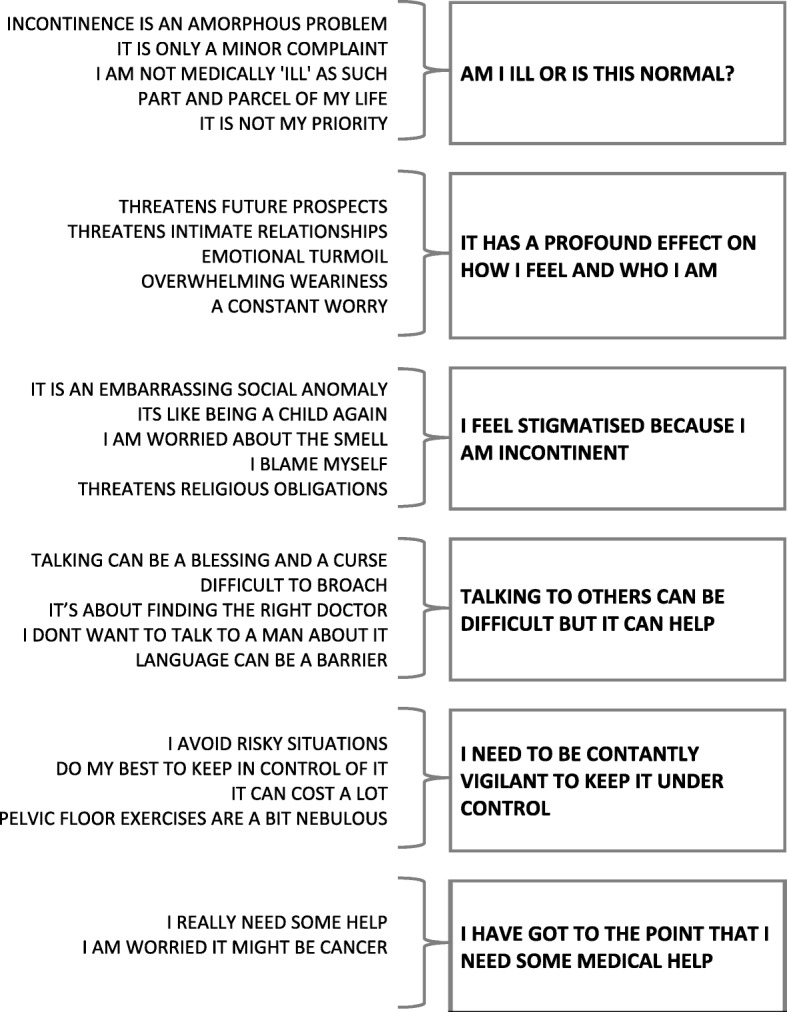
Conceptual categories abstracted into themes, This shows the conceptual categories identified and their abstraction into themes

**Fig. 3:**
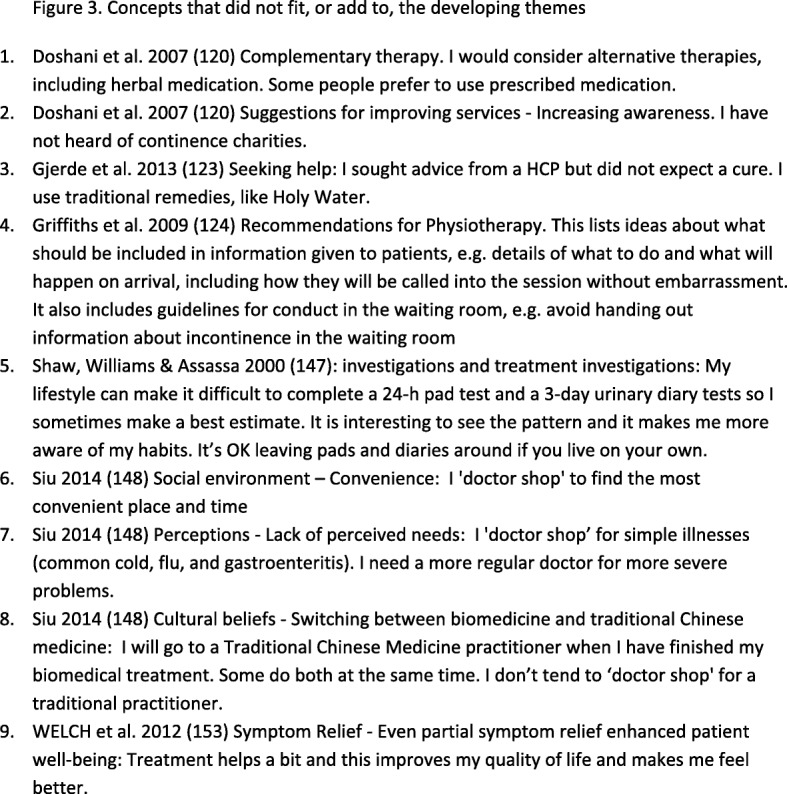
Concepts that did not fit, or add to, the developing themes, This shows the concepts that were identified and not included in the analysis

We organised the concepts into 26 conceptual categories, which we further abstracted into 6 QES findings (Fig. 2): (1) Am I ill or is this normal? (2) It effects who I am and how I feel; (3) I feel stigmatised, ashamed and guilty; (4) talking can be difficult but it can help; (5) keeping incontinence under control; (6) have I got to the point that I need help? Figure 2 shows the abstraction of conceptual categories into themes. There were nine concepts that did not fit or add anything to our analysis (Fig. 3). We describe each of these themes and illustrate them with examples of concepts from the primary studies. These concepts are written in the first person and are not quotations from the primary studies.

### Am I ill or is this normal?

This theme describes the person’s struggle to frame and understand UI (UI). Urination is described as a normal human function, yet also not normal. As such, some described UI as ‘part and parcel of my life’ [142] and (female) inheritance. Accounts describe UI as part of the history of my body and who I am [142] and therefore my burden to bear. People felt that it was normal for mothers and older women to be incontinent.

Peake, Manderson & Potts 1999 [142] - Self-control and the woman out of control: There is no single cause for my incontinence; it is part of the history of my body and who I am. My body is my inheritance. It is normal for older women and mothers to be incontinent. Others are not ‘entitled’ and therefore have to keep it very quiet. I expect to be incontinent because I have children. It is also normal for older women. However, this does not mean that it is socially acceptable to ‘leak’. Leaking is associated with being decrepit and incompetent which makes its secrecy imperative if I am to be seen as normal. It symbolises infancy and extreme old age. It is an embarrassing anomaly.

This struggle to decide whether or not I am ill was exacerbated because UI is as an ‘amorphous problem’ [118]. UI is described as a condition that is not clearly defined without any particular starting point or cause; it is insidious. Accounts describe efforts to explain UI, for example; is it something I have done; is it because I have had children; is it related to another illness; is it because of heavy lifting; is it normal?

Ashworth & Hagan 1993 [118] - Impact of incontinence on daily life: The beginning of incontinence is rarely clear. I have had a problem for a while but only realised in retrospect. There was no clear start point. A distressing accident was the spark which made me aware of the problem. Eventually I realised it was a real problem.

The struggle to understand UI was exacerbated by the thought that you were not *medically* ill. People conceptualised UI as something that was part and parcel of life: it is not a clear, medically accepted and legitimate condition. Some people with UI did not seek help because they felt that the doctor only deals with medical diseases, not ‘personal matters’.

Welch et al. 2012 [153] - Symptom Relief - no treatment as a message that their symptoms were not serious: I have not been offered treatment so it can’t be serious; it must be a normal part of ageing. My tests were negative so I don’t need treatment. It is not a clear, medically accepted and legitimate condition.

People described UI as a minor complaint that was not serious enough to make them go to the doctor.

Ashworth & Hagan 1993 [118]- Incontinence is a vague condition, difficult to grasp - An illegitimate problem: It is not a clear, medically accepted and legitimate condition. It is ‘just a drag’ which interferes with daily life. Is the term ‘incontinence’ too strong to describe what I have? Having ‘the odd squirt’ or ‘leaking’ seems more accurate.

For some, UI was not a priority and other more *serious* conditions and commitments, such as family and work, took precedence.

Hägglund & Wadensten 2007 [125] - Having personal beliefs about seeking care -Toning down their problem: It is not a top priority. Juggling home and family leaves me little time to worry about myself. I am unbearably weary at times. It is not disturbing enough to seek help. Its impact on my job has made me consider discussing it with a nurse.

### It effects who I am and how I feel

Although UI was framed as a normal part of life, the second theme describes its profound effect on a person’s life and sense of self. The constant worry about keeping incontinence under control and concealed from others curtailed peoples’ lives.

Dowd 1991 [121] - Being in Charge - Achieving a normal life means keeping in control of continence through routines: It is a constant concern as continence is unpredictable. Being prepared means I have to be acutely aware of my routine. I need to plan where, when, and with whom I do things. You can be in charge of most situations. If I am not in charge it threatens my self-esteem. If I have an accident I change my routine to increase security. At times I need to drastically alter my routine to prevent accidents and embarrassment. Sometimes I even stay at home and limit social contacts.

For some, UI threatened intimate relationships. Participants described how they no longer felt desirable, and worry about being incontinent during sex had an impact on maintaining current and future relationships.

Peake & Manderson 2003 [141] - Intimate moments: Incontinence affects my sexuality and relationships: I ‘feel guilty,’ ‘disgusting’; ‘something no normal man should have to put up with.’ It makes me anxious when pleasure and evacuation merge. Some partners see it as a strong sexual signal. It is worse to leak at times unrelated to sex. It is particularly problematic when I am trying to make new relationships.

Some felt that UI would have an impact on future prospects and framed it as the harbinger of future frailty: a ‘dark threat on the horizon’ [118]. Participants were afraid of becoming seriously restricted or dependent on others.

Horrocks et al. 2004 [131] - Reactions to incontinence: I am afraid that incontinence is the harbinger of future frailty and dependence. I am worried about cleanliness and personal hygiene. I am embarrassed, ashamed, humiliated, disgusted and so I hide it because I am worried about a negative reaction from other people.

People described emotional turmoil: feeling weepy, distressed, frustrated, annoyed, sad, out of control, fed up; I have lost confidence. For some, hopelessness had made them lose the desire to go on living [127].

Komorowski & Chen 2006 [134] - Impact of UI on quality of life - emotional isolation: I feel alone and excluded. I avoid social activity. I am embarrassed and ashamed, annoyed, frustrated, sad and depressed. It is inconvenient, uncomfortable and dirty. It makes me feel tired. People don’t understand. Other people get used to it and it doesn’t affect them. I am afraid, anxious and lonely. I have lost control.

Some talked about overwhelming weariness from the constant effort to keep UI under control and its effect on sleep.

Nicolson et al. 2008 [140] - The experience of urgency: The experience of urgency has an impact on my quality of life. I need to think about where the loo is. Feeling like I need to go urgency does not necessarily mean that I really need to go. It plays tricks on me. The greatest fear is when I get home and put my key in the lock. My sleep is disturbed and I am exhausted. This makes me feel more anxious and depressed.

### I feel stigmatised, ashamed and guilty

This theme describes UI as a condition that is stigmatised and hidden. As such, those with UI bore the burden of shame. Studies describe UI as an embarrassing social anomaly. Accounts described the stigma that comes from losing control; people did not want to wear the label *incontinent*. UI contravened the normal rules of social etiquette and caused shame and humiliation; it was therefore experienced as a taboo subject. People with UI felt that others would discriminate against them or pass judgement. They described feelings of dirtiness, disgust, shame, embarrassment and humiliation.

Ashworth & Hagan 1993 [118] - Incontinence is a vague condition, difficult to grasp - Incontinence is a forbidden topic: It is not ‘nice’ to talk about incontinence or any ‘things down there’. Normally, people collude politely and don’t mention it. It is a minefield of potential embarrassment. You need permission to break the taboo. Silence protects everyone from it. Even my partner has little or no inkling of the severity of the problem. I wear those panty shield things; can I mention that to you?

Loss of control symbolised infancy (and extreme old age), and some described UI as like being a child again; for example, feeling that: I am an adult and I cannot even control my own body; I have regressed to childhood; I should be responsible for controlling my own body.

Peake, Manderson & Potts 1999 [142] - The Woman in the body: When you are a child growing up, you got into trouble for wetting your pants, and now you are an adult wetting your pants. Wetting yourself is ‘wilful’ or ‘naughty’. It contravenes important childhood messages of body control. It breaches social etiquette: ‘nice’ girls are supposed to ‘smell nice.’ Incontinence is ‘part and parcel’ of women’s lot in life. However, at the same time it ‘makes you feel not the woman you were’.

Loss of control was exacerbated by being worried about the smell:

Griffiths et al. 2009 [124] - Embarrassment: I can tell you some terribly embarrassing stories about times I have wet myself. I worry that I smell. There is a taboo surrounding incontinences. I don’t mention it to anyone, even close family. I am embarrassed to go to the doctor. Not everyone is so embarrassed.

Feeling of blame came hand in hand with the stigma of UI. Although UI was framed as natural part of life, it was a struggle to reconcile this inevitability with a sense of culpability and responsibility. Culpability came with a sense of responsibility for control.

Peake, Manderson & Potts 1999 [142] - Controlling the boundaries of the body: I should be responsible for controlling my own body. I am failing an important social duty. Although it is ‘normal,’ you have a responsibility to find a solution. UI is an artefact of reproduction. It is not fair if you are incontinent and haven’t had children. It makes me feel really dirty. I l have lost self-control. It is difficult to reconcile this sense of self-responsibility with inevitability.

Feelings of guilt focused on personal deficits: for example, people described how they were to blame because they did not ask for help, follow advice or do prescribed exercises.

Ashworth & Hagan 1993 [118] - Effect of incontinence on self-image - sufferers feel that it is their fault: I blame myself because I didn’t do any pelvic floor exercises. I am a bit lazy. I don’t want people to see me buying pads. My health professional is going to tell me off. I am going to be categorised and stigmatised for losing control.

Shame and culpability was exacerbated if they threatened religious obligations, such as prayer. However, there were those that felt that you should not feel shame as it was created by God (Allah).

Van den Muijsenbergh & Lagro-Janssen 2006 [152] - Consequences on daily life: Incontinence has an effect on worship. It is dirty & extremely bothersome. I wash as soon as I can. I always have a supply of pads and clean underwear. Some people talk to their families and partners about it. I am ashamed to talk about it. It has a negative effect on my intimate relationships. I wouldn’t want to remarry because of the incontinence. Some say that you shouldn’t be ashamed because it was created by Allah.

### Talking can be difficult (but it can help)

Although the stigma of UI made it difficult for people to talk about it, this theme describes the recognised benefits of talking to others. Participants described talking as a blessing and a curse [132]. Talking about UI could be a positive experience and it was nice to know you weren’t alone and that UI was normal. Talking could also help people to realise that there were others worse off than you. It can also mean that people can understand and support you. For some, it could help to build close relationships. Talking could also be a cathartic experience and might even help to reduce the taboo.

Hayder & Schnepp 2010 [128] - Regain Control - forming a circle of trust: I find it difficult to talk about my incontinence because I am afraid of negative reactions. I keep it a secret from the outside world. I don’t dare to confide in anyone outside of the family, not even close friends. Because I keep it quite this means I have to miss out on some leisure activities. Other people manage to be very open about it. Talking to someone else who is interested and who understands can create a bond of trust. I would only tell my closest female friends so that they can understand and appreciate my problem. I then wouldn’t have to lie or hide things.

However, participants described the need to manage disclosure of their incontinence with great care.

Ashworth & Hagan 1993 [118] - Incontinence is a vague condition, difficult to grasp - an isolating problem: No one else I know has incontinence like me and I don’t want anyone to discover. I am even careful talking to someone else with a similar problem.

Participants found it difficult to broach the subject of UI with the doctor. They felt that the healthcare professional should enquire as a matter of routine, rather than wait for the patient to initiate the topic.

Hägglund & Wadensten 2007 [125] - Having desired expectations about care - being actively asked about UI: I want my healthcare professional to ask directly about incontinence and for them to create an understanding atmosphere because I don’t dare bring it up.

Some said that it was about finding the right doctor. A ‘match doctor’ [148] was described as more than someone who provides treatment: they hear and respect you; they create an understanding atmosphere by listening and seeing things from your perspective, they are knowledgeable and informative; they respond to individual needs; they are compassionate, empathetic and friendly. Some described how healthcare professionals could be unresponsive, rushed, disinterested, lack of empathy, or even blame you for your problem.

St John James & McKenzie 2002 [151] - Appropriateness and acceptability: I have had negative experiences of healthcare professionals and shop around for the ‘right person’. I want to see someone who is knowledgeable, empathetic, interested in my experience, compassionate, and with whom I feel at ease. I feel grateful to discuss the impact and management of incontinence but it is difficult to raise the issue. For some it is only a minor complaint with little impact.

Language could also be an additional barrier.

Wilkinson 2001 [155] - Communicating and understanding: It is difficult to discuss it in another language and the ‘unresponsiveness’ from healthcare professionals makes this worse. I am too embarrassed to seek help. My healthcare professional is rushed or disinterested. I rely on family or friends to interpret. A bilingual professional would be helpful.

Some women felt that the right healthcare professional would be a woman and that it was s too embarrassing to talk to a man about it [117, 120, 131, 138, 144, 149, 154, 155]. Some were worried about a man performing physical examination. Others felt that UI was a woman’s problem and that men might not be able to see it from a woman’s point of view.

Siu 2015 [149] - Feelings of not being understood: The urologist does not understand the extent of my suffering; they see things from ‘a male’s viewpoint’. I have other things to consider. Treatments can interfere with women’s role and the urologist does not understand this.

### I must keep incontinence under control

This theme describes the need for constant vigilance in order to keep incontinence under control. People planned their routines in advance in order to keep dry and maintain their self-esteem.

This constant worry, and feeling of vulnerability, created a need for various strategies to keep in control of it. Some described how they would rather avoid risky situations. There was a sense of dread about going to places where they didn’t feel safe; they avoided places that they were not familiar with. Some stayed at home and became more and more isolated. Freedom and spontaneity was lost.

Hayder & Schnepp 2010 [128] - A life determined by incontinence: I feel safe when I am at home but dread public areas. I feel vulnerable, ashamed, and abnormal. It affects my relationships and everything that I do. It is difficult to enter into a relationship with a new partner. It has a negative effect on my sexuality. However being able to talk openly about it can help me to build relationships. I am becoming geographically restricted. Trips or journeys into the unknown feel too risky. I feel dependent and inflexible. Over time, I am accepting these limitations and thinking of ways to overcome them.

Some restricted activities, went to the loo much more often and planned their itinerary to fit in with toileting.

Ashworth & Hagan 1993 [118] - Impact of incontinence on daily life - special precautions become a way of life: I have to take more and more precautions. I have to be constantly aware and anticipate leakage. I go to the loo obsessively and plan toilet visits. I check there is a loo. I only do safe activities and avoid some movements. I regulate how much I drink. I choose my clothes carefully. I buy pads in bulk and carry spares. I constantly check for smells and take precautions. Sometimes I throw underwear away.

Strategies to keep UI under control were developed through trial and error and hinged on concealing bodily function, rather than improving or curing it. For example: I wear pads (preferably not incontinence pads); I always know where the loo is and limit drinks.

Getliffe et al. 2007 [122] - Containing the problem: wearing pads doesn’t make you continent, but you need to be confident that a pad will make it invisible to other people. My confidence is fragile and could easily be damaged. I am anxious about leakage. Pad discreteness and odour control is crucial to ‘hiding the problem’. I need to balance absorbency and discreteness (on and in handbag). It also needs to be convenient to change my pad.

Some strategies could incur a cost. For example, pads (and clothing) were expensive.

Milne & Moore 2006 [139] - Barriers to performance of pelvic floor muscle exercises -Financial cost: Continence care is expensive. I am surprised and annoyed that it is not covered by the health care.

In contrast to the strategies that aimed to control through concealment, some used pelvic floor exercises as a strategy for *controlling* incontinence through physical improvement.

Kao et al. 2015 [133] - Developing awareness and gaining control - gaining control and confidence: I pay little attention to pelvic health. My first priority is to my family and I come second. I gained control over my body through pelvic floor exercises and feel more confident.

However, pelvic floor exercises were described as *nebulous* [139], meaning ‘vague, indistinct, formless, ill-defined’ (Oxford English Dictionary online). There was a sense that people needed feedback from the healthcare professional, firstly because it was really difficult to know if you were doing the exercises correctly; and secondly because you did not know if things were improving.

Milne & Moore 2006 [139] - Seeing enhanced believing: If the exercises work I will believe it. I want a test to show that the exercises are working; otherwise it is all a bit nebulous. Changes are very slow and insignificant. It is frustrating and I may give up. If I knew what to expect I could be more realistic about goals.

In this context, some described important competing interests that you need to balance with the effort of doing exercises that have nebulous benefits.

Milne & Moore 2006 [139] - Factors that facilitated pelvic floor muscle exercises - realistic goals and expectations: The exercise regime is very time consuming. You need to be realistic about what you can do in a day.

### Have I got to the point that I need help?

The final theme explores the question - have I got to the point that I need to tell someone about my UI and get some help? This decision was not straightforward and was underpinned by loss of control and intrusion into life. When strategies to conceal or control UI failed and UI started to interfere with life and work, it could get to the point that the need for help outweighed the embarrassment of asking for help.

Ashworth & Hagan 1993 [118] - Impact of incontinence on daily life - dreaded anticipation of worsening in the future: I know that it may sooner or later worsen. I fear being very seriously restricted. I am likely to suffer devastating embarrassment. I will find that over time my strategies will not work, and that I need medical help.

Some sought help because they were worried that UI that it might signify a serious underlying disease, such as cancer.

Shaw et al. 2008 [146] - Identification of the cause of symptoms: I try and find out what is causing this from different sources, like the media. That is how I work out when it is bad enough to seek help. If I know what the cause is, I don’t worry as much. I worried that it might be cancer.

### Conceptual model – living with urinary incontinence: is it just part and parcel of life?

**Fig. 4:**
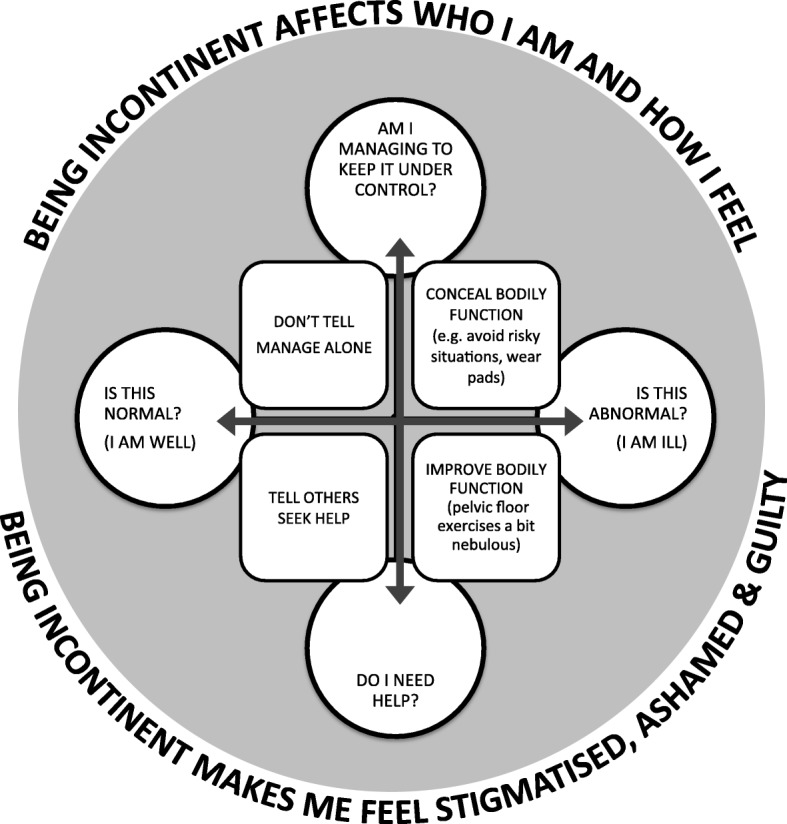
Conceptual model - Living with urinary incontinence: is it just part and parcel of life?**,** Our model shows that being incontinent has an effect on a person’s identity and mood. It conceptualises living with UI as navigating antagonists: (i) Is UI normal or am I ill? (ii) Do I need help or am I managing? (iii) Do I keep UI to myself (and manage alone) or do I tell other people (and get the support that I need)? (iv) Do I use control strategies that focus on *concealing* (avoid risky situations, wear pads) versus, I use strategies that focus on *improving* the bodily function to improve continence. These dualities are not mutually exclusive. Our model highlights the experience of stigma, shame and guilt which exert a pull towards concealment

We developed a conceptual model (Fig. 4) which helps us to understand the challenges of living with UI and some of the barriers to seeking help and receiving appropriate care. Meta-ethnography aims to develop understanding that goes beyond conceptual categories. Our model demonstrates that UI profoundly affects a person’s sense of who they are and also how they feel: it curtails life, affects relationships, and alters future; people feel hopeless and overwhelmed. Our conceptual model highlights antagonists that underpin the experience of UI. Our model illustrates the struggle to decide whether: (i) UI is a normal part of life or I am actually ill and should seek help (ii) I have got to the point that I need help or are things under control? (iii) I keep UI to myself (and manage alone) or do I tell other people (and get the support that I need)? (iv) I use control strategies that focus on *concealing* (avoid risky situations, wear pads) or strategies that focus on *improving* the bodily function to improve continence. Finally our conceptual model highlights the important place of stigma, shame and guilt which are likely to exert a pull towards not telling and concealment of UI.

### Confidence in findings

We assessed our confidence in review findings as moderate (1 finding) to high (5 findings). Details of our GRADE CERQual assessment are shown in Table 4. This assessment of confidence applies only to the 6 QES findings. GRADE-CERQUal was not designed to assess confidence in conceptual models. Two studies identified when we re ran our search did not highlight any additional concepts, suggesting saturation of conceptual categories [168, 169]. We also compared our finding to those in two descriptive QES of UI [25, 26] (Table 5) and found resonance in concepts across studies.

**Table 4:** GRADE-CERQUAL summary of findings

Review finding	Studies contributing	Methodological limitations	Relevance	Coherence number of studies	Adequacy number of themes	Overall confidence
Am i ill or is this normal?	[116–121, 123, 125, 129–131, 133–135, 137–142, 144–146, 150, 151, 153, 154, 156]	3 key paper, 38 satisfactory	34 direct, 3 indirect, 3 partial	28/41	80	High 20 to 31 studies contributed 69 to 93 concepts that supported these themes. We also found rich use of narrative to support the themes
It effects who i am and how i feel	[126, 127, 129–131, 133–137, 139, 141–145, 148, 149, 151, 153]	2 key paper, 39 satisfactory	37 direct, 3 indirect 1 partial	20/41	69	
I feel stigmatised, ashamed and guilty	[116–119, 121–125, 127–131, 134–137, 140–145, 149, 152, 155, 156]	4 key paper, 37 satisfactory	38 direct, 2 indirect 1 partial	28/41	80	
Talking can be difficult (but it can help)	[116–120, 122–125, 128–134, 137, 138, 140, 142, 144–149, 151, 153–156]	3 key paper, 38 satisfactory	33 direct, 5 indirect, 3 partial	31/41	93	
I must keep incontinence under control	[116–123, 125, 127, 128, 131, 133–143, 150–153, 155, 156]	4 key paper, 37 satisfactory	34 direct, 4 indirect, 3 partial	29/41	76	
Have i got to the point that i need help?	[118, 125, 134, 135, 145, 146, 150, 154, 155]	1 key paper, 40 satisfactory	40 direct, 1 indirect	9/41	17	Moderate downgraded due to fewer studies contributing although narrative rich

**Table 5:** translation of ideas across QES

Original Finding	Essence Of Original Finding Translated Into First Person	Am i ill or is this normal	It effects who i am and how i feel	I feel stigmatised, ashamed & guilty	Talking can be difficult (but it can help)	I must keep it under control	Have i got to the point that i need help?
Mendes & Colleague s[26]: Cultural and religious backgrounds and personal reluctance contribute to delays in seeking UI treatment	I don’t want to ask for professional help. There is no opportunity to share my problems with a HCP, I want to deal with it without professional help, I don’t have a clear physical symptom; I don’t want to find out that I have something serious; God will cure me; he won’t let me suffer. It’s not serious; other things are more important	YES					YES
Mendes & Colleague s[26]: The inevitable and regrettable problem of UI endured silently and alone affects women’s daily activities and their social roles	I regret having UI; but other people have it too. My daily life is affected. I avoid thing that I used to do; I don’t go to RE and social gatherings as much now I silently endure the loneliness. I can’t go out because I need the loo all the time. I worry about what others will think and keep it a secret.		YES	YES	YES	YES	
Mendes & Colleague s[26]: Poor knowledge and the vague nature of the symptoms mask the fact that UI is a disease	UI is a vague condition that we don’t know much about. The symptoms are vague. It is not recognised as a disease. We don’t talk about it so people don’t know about it.	YES					
Mendes & Colleagues [26]: The experiences provoked by UI and the sense of shame regarding the condition have impaired women’s lives	UI makes me anxious, distressed. It carries a stigma. It is so embarrassing and humiliating when I leak in public. I feel depressed and hopeless and have low self-esteem because I can’t do the things that I used to do and I can’t do much to help myself. It is an emotional experience. I am ashamed	YES		YES			
Mendes & Colleague s[26]: UI has provoked negative effects on women’s intimacy and sexual satisfaction and provoked changes in the way they experience their sexuality and sexual function	I feel fear, shame, blame and guilt. It has affected intimacy and my sexual satisfaction.	YES		YES			
Mendes & Colleague s[26]: UI is considered a consequence of pregnancy and childbirth, inherent to aging or a religious punishment	UI is a natural result of pregnancy and childbirth. It is part of ageing. It is a punishment and I must just accept it.	YES					
Mendes & Colleague s[26]: The women affected by UI adopt several strategies to improve their health status	I try and learn things so that I can control the consequences of UI. I seek professional help. I try and keep it under control; I manage using various strategies					YES	YES
Mendes & Colleague s[26]: Women have personal preferences towards care providers and treatments; they confront difficulties through UI treatment and some care needs are not met’	My health needs are not being met. Doctors lack interest. I am not being taken seriously. There are language barriers; there is no medical diagnosis or plan. It is difficult to understand /adhere to treatments prescribed. I prefer a female Hcp; I prefer my own family to translate or at least someone of my own ethnicity				YES		
Siddiqui and Colleagues [25]: UI management	I need to adhere to strict routines and strategies to manage UI. I need to make plans for being in public, including knowing where toilets are. It is only a minor, it is normal so I don’t seek treatment. It is difficult to communicate with the dr. they don’t take it seriously. They need to talk more clearly. I would prefer to see a woman	YES			YES	YES	
Siddiqui and Colleagues [25]: UI experience	I feel fear, stigmatization, and shame. I worry what people think. I feel disgusting.		YES	YES			

## Discussion

Qualitative research is interpretive and the researcher’s perspectives can influence findings. FT (female, aged 51) is an anthropologist, qualitative researcher with experience in QES, and qualified physiotherapist with an interest in chronic pain. KB (female, aged 56) is clinical director of a UK, NHS musculoskeletal directorate and qualified physiotherapist. Their interest in UI comes from the desire to improve services for patients with musculoskeletal disorders who often experience UI. They also have a methodological interest in conceptual QES.

We developed six conceptual categories: (1) Am I ill or is this normal? (2) It effects who I am and how I feel; (3) I feel stigmatised, ashamed and guilty; (4) talking can be difficult but it can help; (5) keeping incontinence under control; (6) have I got to the point that I need help? These categories can help us to understand what it is like to live with UI, and also to understand the barriers to healthcare. In particular, the difficulty of deciding whether or not incontinence is a medical problem the stigma of being incontinent, finding it difficult to talk about incontinence and also the use of effective strategies to stay dry (pads, staying at home, micromanaging access to toilets) can all be barriers to seeking help and accessing strategies to improve continence.

Our findings demonstrate that even though UI is framed as part and parcel of life (up to a point); it profoundly affects a person’s quality of life. Our findings demonstrate the complexity of: deciding whether or not I am ill enough to seek help; deciding whether to tell someone or to keep quiet; deciding to use strategies that conceal UI or strategies (although nebulous) that might make me continent again. These decisions pivot upon the success (or not) of keeping UI *‘under control’.* Our model demonstrates that it is not always clear whether or not I am ill, and whether or not I should seek help. Similarly, in a meta-ethnography of chronic pelvic pain, Toye, Seers and Barker found that women with chronic pelvic pain struggled to ‘know if my pain is abnormal or whether this is normal for women’ [14].

Our findings also indicate that the challenge of accessing healthcare is exacerbated by a *culture of secrecy* around certain conditions, such as UI and chronic pelvic pain [14]: Incontinence is experienced as taboo. Talking about UI can be a *blessing and a curse* [132], and needs to be carefully managed. Although there are clear benefits to be gained from discussing UI, the risk of exposure can be a weighty burden to bear. We see that people with UI feel stigmatised, ashamed and guilty. Healthcare encounters can contribute to this sense of guilt: for example, at times patients are made to feel that UI ‘is my fault because I haven’t done my exercises’. It can be difficult to reconcile the idea that incontinence is a normal and expected part of life with these associated feelings of shame: ‘I feel vile’.

There are anthropological concepts that help to explain the profound sense of defilement associated with UI. For example, in the seminal text, ‘Purity and Danger’, Douglas explores the concept of pollution and taboo [170]. Douglas describes how feelings of abhorrence, defilement and ‘dirt’ stem from a cultural reaction to ambiguity and anomaly: ‘Dirt offends against order’ (page 2). Our findings demonstrate that UI is indeed experienced as anomalous, as it defies social categorisation: I am ill and also not ill; urination is normal and also not normal; I am an adult and also not like an adult. This sense of anomaly is intensified because the boundary between inside and outside the body is unconventionally and unexpectedly breached. Thus, people living with UI exist in a liminal social space that is ‘betwixt and between’ states [171]:

‘These [liminal] persons elude or slip through the network of classifications that normally locate states and positions in cultural space. Liminal entities are neither here nor there; they are betwixt and between the positions assigned and arrayed by law, custom, convention, and ceremonial’ (page 144).

The anomaly of UI can make it very difficult talk about, and this may help to explain why only the tip of the iceberg seek care or see a pelvic floor specialist [4]. Although Talcott-Parson’s *Sick Role* [172] indicates that we are obliged to go to the doctor in order to access the privileges of the Sick Role, people with ambiguous or stigmatised conditions are torn between the incentive to access these privileges, and the cultural incentive to keep quiet.

Our conceptual model also highlights potential differences between patients and healthcare professionals’ understanding of what it means to keep UI under control. We see that people with UI focus on *concealing* UI, rather than improving continence, and go to great lengths to avoid risky situations. In contrast to this, the NICE guidelines for incontinence recommend PFMT as the first line treatment for UI, and stipulate that concealment strategies such as, ‘absorbent products, hand held urinals and toileting aids should not be considered as a treatment for UI’, but only used as ‘a coping strategy pending definitive treatment an adjunct to ongoing therapy’ [173]. Although a Cochrane review of trials found that PFMT can ‘cure and improve’ UI [3], our finding show that people find exercises somewhat nebulous. This is likely to be a significant barrier to adherence for PFMT. Hay-Smith and colleagues also found that women with UI could find it difficult to understand and perform PFMT [23].

Toye and colleagues suggest that it would be ‘useful to find ways in which we can be more discerning about what to include’ in QES [35]. QES reviewers spend a lot of time searching for studies and this time might be more valuable spent on analysis. Toye and colleagues suggest that it is important for reviewers (and funders) to consider whether or not an exhaustive search of the literature is necessary for QES [8]. We limited our search to Medline for several reasons: firstly, previous QES have shown that Medline retrieves a large proportion of the studies included in a QES [7, 13, 14, 35]; secondly, our scoping review indicated that we were likely to find a large number of studies; thirdly, although Campbell and colleague suggest that 38 studies is sufficient for meta-ethnography [33], there is limited agreement about the optimum amount of studies required to have confidence in QES findings. Intuitively, a certain weight and spread of data appears more convincing. However, the *richness* of data contributing to a finding may make a weightier contribution to idea development. Our findings raise some concerns about the comprehensiveness of search strategies for QES. Firstly, our search of a single database using search terms developed for QES identified more studies than other QES using more than one database. Secondly, other QES exploring particular aspects of UI indicate that there are other relevant studies that were not found; for example [62, 174–177]. Finally, through serendipity we identified other relevant studies beyond our purposive search; for example [178, 179]. The question remains, how long is a piece of string? It may be that continuing to search for studies beyond the point of theoretical saturation is not time well spent. The two studies identified when we re ran our search did not highlight any additional concepts, suggesting saturation of conceptual categories [168, 169]. Our findings resonate with those in two descriptive QES of UI [25, 26]. Siddiqui and colleagues discuss the shame and secrecy of UI (experiences), the struggle to decide whether it is normal and if anything will help (understanding), the secrecy and difficulty communicating with health professionals (behaviours). Vetthanayagam and colleagues also explore ‘being brushed aside’ by health professionals, the feeling that I must ‘put up with it’ because it is normal and because I am embarrassed and also the point at which ‘something has to be done’.

A growing number of researchers are appraising studies for QES; the percentage of qualitative syntheses including quality appraisal increased from 40% (1988–2004) to 72% (2005–2008) [18]. This number may increase further in the current research climate. However, Campbell and colleagues [33] argue that ‘inclusion of poorer quality qualitative research. .. is unlikely to be as damaging’ and do not exclude studies on the basis of poorly reported methods [33]. In contrast, Dixon Woods and colleagues exclude studies which they judge to be ‘fatally flawed’ [17]. We made the cost-benefit decision to appraise studies, as suggested by Dixon-Woods, as: ‘key’ (conceptually rich); ‘satisfactory’; ‘irrelevant’; or ‘fatally flawed’ [37]. For the purposes of meta-ethnography, we suggest that it is important to determine whether or not a study is good enough or *satisfactory* [38]. However, high quality studies that include robust ideas will influence the analytical output and weaker studies will not: thus we agree that low quality studies will do no harm. Similarly, no harm is done by including themes from studies that draw data from the same cohort. This is a key difference between meta-analysis and meta-ethnography. This is because QES relies on abstracting the essence of ideas from studies, and it is very common for different themes from the same cohort to appear in different publications. This does no harm because QES does not hinge on numerical analysis.

We aimed to use QES to further understand the experience of living with UI and the potential barriers to seeking help and therefore receiving appropriate treatment. Most of the studies explored women’s experience of UI and further studies might usefully explore, and compare, what it is like for men. We did not include the experience of pregnant women or the experience within the first year peri-partum. However, we did include two studies by Mason and colleagues [137, 138] as these included the experience of 15 women who were symptomatic 1 year after childbirth. However, the first experience of involuntary leakage for many women is during pregnancy or after a birth, and childbirth is a strong predictor of UI. Further research would usefully explore the differences is experience during childbirth and peri-partum. The majority of studies explored experience of women in high income countries; only four studies explored the experience of women from in upper middle income countries (Brazil, China and Iran) and one study explored the experience in a lower income country (Ethiopia) (Table 2). It is likely that the experience of incontinence would differ between countries and further research might highlight these differences. However, we demonstrate that there are important themes that cut across age, gender and type of UI. Further primary studies would usefully explore differences in specific samples related to gender, age, pathophysiology or other factors.

## Conclusions

There are some important clinical considerations and implications that can be drawn. Firstly, the culture of secrecy and profound sense of shame felt by those with UI makes it very difficult for people to talk about it and to seek help. Proactively asking patients about UI would help to reduce the taboo and ensure that help is made available. This finding may be usefully extended to other anomalous and embarrassing health conditions. Secondly, differences in the concept of what it is to keep UI under control have direct implications for clinical care. Research indicates that PFMT can improve continence and yet this is not the focus of many people’s control strategies. A cultural environment which reduces the shame and stigma of UI may help people to switch the focus to strategies that will improve continence (i.e. PFMT). At the same time, it would be useful for healthcare professionals to recognise a person’s need to conceal UI, and to collaborate in a treatment approach that not only facilitates this need, but that also aims to improve continence. Finally, future research should address the issue of nebulosity of PFMT, as this is likely to have a direct effect on adherence when balanced against a person’s competing needs.

## Data Availability

This is a large body of qualitative data translated from published primary qualitative studies. The first person translations of this primary research are available from the corresponding author on reasonable request.

## Abbreviations

GRADE-CERQual: Grading of Recommendations Assessment, Development and Evaluation-Confidence in the Evidence from Reviews of Qualitative research
NHS: National Health Service (UK)
PFMT: Pelvic floor muscle training
QES: Qualitative Evidence Synthesis
STARLITE: Sampling strategy, type of study, approaches, range of years, limits, inclusion and exclusions, terms used, electronic sources
UI: Urinary Incontinence

## References

[1] Hay-Smith E, Herderschee R, Dumoulin C, Herbison G (2011). Comparisons of approaches to pelvic floor muscle training for urinary incontinence in women (Review). Cochrane Database Syst Rev.

[2] Buckley BS, Lapitan MCM (2010). Prevalence of urinary incontinence in men, women, and children—current evidence: findings of the fourth international consultation on incontinence. Urology..

[3] Dumoulin C, Cacciari LP, Hay-Smith EJC (2018). Pelvic floor muscle training versus no treatment, or inactive control treatments, for urinary incontinence in women. Cochrane Database Syst Rev..

[4] Minassian V, Yan X, Lichtenfeld M, Sun H, Stewart W (2012). The iceberg of health care utilization in women with urinary incontinence. Int Urogynecol J..

[5] Noyes J, Popay J, Pearson A, Hannes K, Booth A, Higgins J, Green S (2008). Qualitative research and cochrane reviews. Cochrane Handbook for Systematic Reviews.

[6] Noblit G, Hare R (1988). Meta-ethnography: Synthesising qualitative studies.

[7] Toye F, Seers K, Allcock N, Briggs M, Carr E, Andrews J, Barker K (2013). A meta-ethnography of patients' experiences of chronic non-malignant musculoskeletal pain. Health Serv Deliv Res..

[8] Toye F, Seers K, Allcock N, Briggs M, Carr E, Barker K (2014). Meta-ethnography 25 years on: challenges and insights for synthesising a large number of qualitative studies. BMC Med Res Methodol..

[9] France EF, Cunningham M, Ring N, Uny I, Duncan EA, Jepson RG, Maxwell M, Roberts RJ, Turley RL, Booth A, et al. Improving reporting of meta-ethnography: The eMERGe reporting guidance. J Adv Nurs. 0(0) p. 1-13. 10.1111/jan.13809 PMC7594209 30644123

[10] Toye F, Seers K, Allcock N, Briggs M, Carr E, Barker K (2016). A synthesis of qualitative research exploring the barriers to staying in work with chronic musculoskeletal pain. Disabil Rehabil..

[11] Daker-White G, Donovan J, Campbell R (2014). Redefined by illness: meta-ethnography of qualitative studies on the experience of rheumatoid arthritis. Disabil Rehabil..

[12] Mengshoel A, Marit A, Sim J, Birgitte A, Madden S. Diagnostic experience of patients with fibromyalgia - A meta-ethnography. Chronic Illn. 2017;0(0) http://journals.sagepub.com/doi/pdf/10.1177/1742395317718035. 10.1177/1742395317718035 28762775

[13] Barker K, Toye F, Lowe C. A qualitative systematic review of patients' experience of osteoporosis using meta-ethnography. Arch Osteoporos. 2016;11(1):1–13. 10.1007/s11657-016-0286-z PMC5063904 27739032

[14] Toye F, Seers K, Barker K (2014). A meta-ethnography of patients' experiences of chronic pelvic pain: struggling to construct chronic pelvic pain as ‘real’. J Adv Nurs..

[15] Sandelowski M, Barrosso J (2007). Handbook for synthesising qualitative research.

[16] Dixon-Woods M, Booth A, Sutton A (2007). Synthesizing qualitative research: a review of published reports. Qual Res..

[17] Dixon-Woods M, Agarwal S, Jones D, Young B, Sutton A (2005). Synthesising qualitative and quantitative research evidence: a review of possible methods. J Health Serv Res Policy..

[18] Hannes K, Macaitis K (2012). A move to more systematic and transparent approaches in qualitative evidence synthesis: update on a review of published papers. Qual Res..

[19] Barnett-Page E, Thomas J (2009). Methods for synthesis of qualitative research: a critical review. Economic and Social Research Council Research Methods, National Centre for Research Methods Working Paper Series (01/09).

[20] Toye F, Seers K, Hannink E, Barker K (2017). A mega-ethnography of eleven qualitative evidence syntheses exploring the experience of living with chronic non-malignant pain. BMC Med Res Methodol..

[21] Toye F, Seers K, Barker K: Living life precariously with rheumatoid arthritis - A mega-ethnography of nine qualitative evidence syntheses. BMC Rheumatol 2019, accepted for publication**.** 10.1186/s41927-018-0049-0 PMC6390589 30886993

[22] French B, Thomas LH, Harrison J, Coupe J, Roe B, Booth J, Cheater FM, Leathley MJ, Watkins CL, Hay-Smith J (2017). Client and clinical staff perceptions of barriers to and enablers of the uptake and delivery of behavioural interventions for urinary incontinence: qualitative evidence synthesis. J Adv Nurs..

[23] Hay-Smith J, Dean S, Burgio K, McClurg D, Frawley H, Dumoulin C (2015). Pelvic-floor-muscle-training adherence ‘modifiers’: a review of primary qualitative studies—2011 ICS state-of-the-science seminar research paper III of IV. Neurourol Urodyn..

[24] Avery JC, Braunack-Mayer AJ, Stocks NP, Taylor AW, Duggan P (2013). Psychological perspectives in urinary incontinence: a metasynthesis. OA Women’s Health..

[25] Siddiqui N, Levin P, Ammarell N, Phadtare A, Pietrobon R (2012). Perceptions about female urinary incontinence in different racial and ethnic groups: a systematic review. Neurourol Urodyn..

[26] Mendes A, Hoga L, Goncalves B, Silva P, Pereira P (2017). Adult women's experiences of urinary incontinence: a systematic review of qualitative evidence. JBI Database System Rev Implement Rep..

[27] Wong SSWN, Haynes RB (2004). Developing optimal search strategies for detecting clinically relevant qualitative studies in MEDLINE. Medinfo..

[28] Wilczynski NLMS, Haynes RB (2007). Search strategies for identifying qualitative studies in CINAHL. Qual Health Res..

[29] McKibbon KAWN, Haynes RB (2006). Developing optimal search strategies for retrieving qualitative studies in PsycINFO. Eval Health Prof..

[30] Haynes RB, WN WLA (2006). Hedges team. Developing optimal search strategies for retrieving clinically relevant qualitative studies in EMBASE. Qual Health Res..

[31] Toye F, Seers K, Barker K (2018). A meta-ethnography of health-care professionals' experience of treating adults with chronic non-malignant pain to improve the experience and quality of health care. In: Health Services and Delivery Research. Edn.

[32] Barker K, Toye F, MinnsLowe C. A qualitative systematic review of patients' experience of osteoporosis using meta-ethnography. Arch Osteoporos. 2016;11(33) 10.1007/s11657-11016-10286-z. 10.1007/s11657-016-0286-z PMC5063904 27739032

[33] Campbell R, Pound P, Morgan M, Daker-White G, Britten N, Pill R, Yardley L, Pope C, Donovan J (2011). Evaluating meta-ethnography: systematic analysis and synthesis of qualitative research. Health Technol Assess..

[34] Charmaz K (2006). Constructing grounded theory.

[35] Toye F, Seers K, Tierney S, Barker K (2017). A qualitative evidence synthesis to explore healthcare professionals’ experience of prescribing opioids to adults with chronic non-malignant pain. BMC Fam Pract..

[36] Booth A (2006). ‘Brimful of STARLITE’: toward standards for reporting literature searches. J Med Libr Assoc..

[37] Dixon-Woods M, Sutton A, Shaw R, Miller T, Smith J, Young B, Bonas S, Booth A, Jones D (2007). Appraising qualitative research for inclusion in systematic reviews: a quantitative and qualitative comparison of three methods. J Health ServRes Policy..

[38] Toye F, Seers K, Allcock N, Briggs M, Carr E, Andrews J, Barker K (2013). 'Trying to pin down jelly' - exploring intuitive processes in quality assessment for meta-ethnography. BMC Med Res Methodol..

[39] Nvivo (2010). Nvivo qualitatitve data analysis and software, QSR International Pty Ltd. Software for qualitative data analysis.

[40] Tavory I, Timmermans S (2014). Abductive analysis - theorizing qualitative research.

[41] Pope C, Mays N, Popay J (2007). Synthesizing qualitative and quantitative health research: a guide to methods.

[42] Lewin S, Glenton C, Munthe-Kaas H, Carlsen B, Colvin CJ, Gülmezoglu M, Noyes J, Booth A, Garside R, Rashidian A (2016). Using Qualitative Evidence in Decision Making for Health and Social Interventions: An Approach to Assess Confidence in Findings from Qualitative Evidence Syntheses (GRADE-CERQual). PLOS Med..

[43] Noyes J, Booth A, Lewin S, Carlsen B, Glenton C, Colvin CJ, Garside R, Bohren MA, Rashidian A, Wainwright M (2018). Applying GRADE-CERQual to qualitative evidence synthesis findings–paper 6: how to assess relevance of the data. Implement Sci..

[44] Munthe-Kaas H, Bohren M, Glenton C, Lewin S, Noyes J, Tunçalp Ö, Booth A, Garside R, Colvin C, Wainwright M (2018). Applying GRADE-CERQual to qualitative evidence synthesis findings—paper 3: how to assess methodological limitations. Implement Sci..

[45] Lewin S, Booth A, Glenton C, Munthe-Kaas H, Rashidian A, Wainwright M, Bohren M, Tunçalp Ö, Colvin C, Garside R (2018). Applying GRADE-CERQual to qualitative evidence synthesis findings: introduction to the series. Implement Sci..

[46] Lewin S, Bohren M, Rashidian A, Munthe-Kaas H, Glenton C, Colvin C, Garside R, Noyes J, Booth A, Tunçalp Ö (2018). Applying GRADE-CERQual to qualitative evidence synthesis findings—paper 2: how to make an overall CERQual assessment of confidence and create a Summary of Qualitative Findings table. Implement Sci..

[47] Glenton C, Carlsen B, Lewin S, Munthe-Kaas H, Colvin CJ, Tunçalp Ö, Bohren MA, Noyes J, Booth A, Garside R (2018). Applying GRADE-CERQual to qualitative evidence synthesis findings—paper 5: how to assess adequacy of data. Implement Sci..

[48] Colvin C, Garside R, Wainwright M, Munthe-Kaas H, Glenton C, Bohren M, Carlsen B, Tunçalp Ö, Noyes J, Booth A (2018). Applying GRADE-CERQual to qualitative evidence synthesis findings—paper 4: how to assess coherence. Implement Sci..

[49] Booth A, Lewin S, Glenton C, Munthe-Kaas H, Toews I, Noyes J, Rashidian A, Berg RC, Nyakang’o B, Meerpoh JJ (2018). Applying GRADE-CERQual to qualitative evidence synthesis findings–paper 7: understanding the potential impacts of dissemination bias. Implement Sci..

[50] Kemp K, Griffiths J, Lovell K (2012). Understanding the health and social care needs of people living with IBD: a meta-synthesis of the evidence. World J Gastroenterol..

[51] Koch LH (2006). Help-seeking behaviors of women with urinary incontinence: an integrative literature review. J Midwifery Women’s Health..

[52] Mendes A, Hoga L, Gonçalves B, Silva P, Pereira P (2015). Adult women's experiences of urinary incontinence: a systematic review of qualitative evidence protocol. JBI Database System Rev Implement Rep..

[53] Ostaszkiewicz J, O'Connell B, Dunning T (2012). Residents' perspectives on urinary incontinence: a review of literature. Scand J Caring Sci..

[54] Strickland R (2014). Reasons for not seeking care for urinary incontinence in older community-dwelling women: a contemporary review. Urol Nurs..

[55] Terzoni S, Montanari E, Mora C, Destrebecq A (2011). Urinary incontinence in adults: nurses' beliefs, education and role in continence promotion. A narrative review. Arch Ital Urol Androl..

[56] Dingwall L, McLafferty E (2006). Do nurses promote urinary continence in hospitalized older people?: an exploratory study. J Clin Nurs..

[57] Ehlman K, Wilson A, Dugger R, Eggleston B, Coudret N, Mathis S (2012). Nursing home staff members' attitudes and knowledge about urinary incontinence: the impact of technology and training. Urol Nurs..

[58] Lyons SS (2010). How do people make continence care happen? An analysis of organizational culture in two nursing homes. Gerontologist..

[59] MacDonald CD, Butler L (2007). Silent no more: elderly women's stories of living with urinary incontinence in long-term care. J Gerontol Nurs..

[60] Mason M, Tully S (2002). Urinary incontinence in the older acute care population: effects of knowledge, attitudes and beliefs of nurses on continence management. Perspectives..

[61] Mather KF, Bakas T (2002). Nursing assistants' perceptions of their ability to provide continence care. Geriatr Nurs (New York, NY).

[62] O'Dell KK, Jacelon C, Morse AN (2008). 'I'd rather just go on as I am'--pelvic floor care preferences of frail, elderly women in residential care. Urol Nurs..

[63] Resnick B, Keilman LJ, Calabrese B, Parmelee P, Lawhorne L, Pailet J, Ouslander J (2006). Nursing staff beliefs and expectations about continence care in nursing homes. J Wound Ostomy Continence Nurs..

[64] Robinson JP (2000). Managing urinary incontinence in the nursing home: residents' perspectives. J Adv Nurs..

[65] Sylvia CJ, Jones V (2010). The lived experience of the wound, ostomy, and continence nurse in wound care. J Wound Ostomy Continence Nurs..

[66] Taunton RL, Swagerty DL, Lasseter JA, Lee RH (2005). Continent or incontinent? That is the question. J Gerontol Nurs..

[67] Chelvanayagam S, Stern J (2007). Using therapeutic groups to support women with faecal incontinence. Br J Nurs.

[68] Collings S, Norton C (2004). Women's experiences of faecal incontinence: a study. Br J Community Nurs..

[69] Frohlich DO (2014). Support often outweighs stigma for people with inflammatory bowel disease. Gastroenterol Nurs..

[70] Olsson F, Berterö C (2015). Living with faecal incontinence: trying to control the daily life that is out of control. J Clin Nurs..

[71] Peden-McAlpine C, Bliss D, Becker B, Sherman S (2012). The experience of community-living men managing fecal incontinence. Rehabil Nurs..

[72] Peden-McAlpine C, Bliss D, Hill J (2008). The experience of community-living women managing fecal incontinence. West J Nurs Res..

[73] Rasmussen JL, Ringsberg KC (2010). Being involved in an everlasting fight--a life with postnatal faecal incontinence. A qualitative study. Scand J Caring Sci..

[74] Rimmer CJ, Gill KA, Greenfield S, Dowswell G (2015). The design and initial patient evaluation of an integrated care pathway for faecal incontinence: a qualitative study. BMC Health Serv Res..

[75] Tucker J, Clifton V, Wilson A (2014). Teetering near the edge; women's experiences of anal incontinence following obstetric anal sphincter injury: an interpretive phenomenological research study. Aust N Z J Obstet Gynaecol..

[76] Wilson M (2007). The impact of faecal incontinence on the quality of life. Br J Nurs.

[77] Wilson M (2013). Living with faecal incontinence: follow-up to a research project. Br J Nurs.

[78] Wilson M (2015). Living with faecal incontinence: a 10-year follow-up study. Br J Nurs.

[79] Brittain KR, Shaw C (2007). The social consequences of living with and dealing with incontinence--a carers perspective. Soc Sci Med (1982).

[80] Cassells C, Watt E (2003). The impact of incontinence on older spousal caregivers. J Adv Nurs..

[81] Coyne KS, Matza LS, Brewster-Jordan J (2009). ‘We have to stop again?!’: The impact of overactive bladder on family members. Neurourol Urodyn..

[82] Hayder D, Schnepp W (2008). Urinary incontinence - the family caregivers’ perspective. Z Gerontol Geriatr..

[83] Santini S, Andersson G, Lamura G (2016). Impact of incontinence on the quality of life of caregivers of older persons with incontinence: a qualitative study in four European countries. Arch Gerontol Geriatr..

[84] Lobchuk MM, Rosenberg F (2014). A qualitative analysis of individual and family caregiver responses to the impact of urinary incontinence on quality of life. J Wound Ostomy Continence Nurs..

[85] Butterfield YC, O'Connell B, Phillips D (2007). Peripartum urinary incontinence: a study of midwives' knowledge and practices. Women Birth..

[86] Grealish M, O'Dowd TC (1998). General practitioners and women with urinary incontinence. Br J General Pract..

[87] Hägglund D (2010). District continence nurses' experiences of their continence service in primary health care. J Nurs Manag..

[88] Hutchings J, Sutherland L (2014). Student nurse understanding of the psychosocial impact of urinary incontinence. Urol Nurs..

[89] Newman DK (2009). Talking to patients about bladder control problems. Nurse Pract..

[90] Peters S (1997). Don't ask, don't tell. Breaking the silence surrounding female urinary incontinence. Adv Nurse Pract..

[91] Rolnick S, Bliss DZ, Jackson JM, Arntson C, Mullins J, Hepburn K (2013). Healthcare providers' perspectives on communicating incontinence and skin damage information with patients with dementia and their family caregivers: a descriptive study. Ostomy Wound Manage..

[92] Shaw C, Atwell C, Wood F, Brittain K, Williams K (2007). A qualitative study of the assessment and treatment of incontinence in primary care. Fam Pract..

[93] Siu JY-M (2015). Communicating with mismatch and tension: treatment provision experiences of primary care doctors treating patients with overactive bladder in Hong Kong. BMC Fam Pract..

[94] Tannenbaum C, Labrecque D, Lepage C (2005). Understanding barriers to continence care in institutions. Can J Aging..

[95] Teunissen D, van den Bosch W, van Weel C, Lagro-Janssen T (2006). Urinary incontinence in the elderly: attitudes and experiences of general practitioners. A focus group study. Scand J Prim Health Care..

[96] Basu M, Duckett JRA (2009). Barriers to seeking treatment for women with persistent or recurrent symptoms in urogynaecology. BJOG..

[97] Sevilla C, Wieslander CK, Alas AN, Dunivan GC, Khan AA, Maliski SL, Rogers RG, Anger JT (2013). Communication between physicians and Spanish-speaking Latin American women with pelvic floor disorders: a cycle of misunderstanding?. Female Pelvic Med Reconstr Surg..

[98] Srikrishna S, Robinson D, Cardozo L (2009). Qualifying a quantitative approach to women's expectations of continence surgery. Int Urogynecol J Pelvic Floor Dysfunct..

[99] Buurman MBR, Lagro-Janssen ALM (2013). Women's perception of postpartum pelvic floor dysfunction and their help-seeking behaviour: a qualitative interview study. Scand J Caring Sci..

[100] Roe B, May C (1999). Incontinence and sexuality: findings from a qualitative perspective. J Adv Nurs..

[101] Lindeman K, Li Y, Palmer MH (2012). Help-seeking for incontinence by individuals with heart failure. J Am Geriatr Soc..

[102] Bush TA, Castellucci DT, Phillips C (2001). Exploring women's beliefs regarding urinary incontinence. Urol Nurs..

[103] Bradway C, Dahlberg B, Barg FK (2010). How women conceptualize urinary incontinence: a cultural model. J Women’s Health (2002).

[104] Hart KJ, Palmer MH, Fitzgerald S (1999). Perceived causes of urinary incontinence and reporting: a study with working women. Clin Nurs Res..

[105] Chaliha C, Stanton SL (1999). The ethnic cultural and social aspects of incontinence--a pilot study. Int Urogynecol J Pelvic Floor Dysfunct..

[106] Kang Y, Crogan NL (2008). Social and cultural construction of urinary incontinence among Korean American elderly women. Geriatr Nurs (New York, NY).

[107] Anger JT, Le TX, Nissim HA, Rogo-Gupta L, Rashid R, Behniwal A, Smith AL, Litwin MS, Rodriguez LV, Wein AJ (2012). How dry is ‘OAB-dry’? Perspectives from patients and physician experts. J Urol..

[108] Coyne KS, Sexton CC, Thompson C, Bavendam T, Brubaker L (2015). Development and psychometric evaluation of the urgency questionnaire for evaluating severity and health-related quality of life impact of urinary urgency in overactive bladder. Int Urogynecol J..

[109] Bradway C, Strumpf N (2008). Seeking care: women's narratives concerning long-term urinary incontinence. Urol Nurs..

[110] Bradway CW, Barg F (2006). Developing a cultural model for long-term female urinary incontinence. Soc Sci Med (1982).

[111] Bradway C (2005). Women's narratives of long-term urinary incontinence. Urol Nurs..

[112] Diokno AC, Sand PK, Macdiarmid S, Shah R, Armstrong RB (2006). Perceptions and behaviours of women with bladder control problems. Fam Pract..

[113] Filipetto FA, Fulda KG, Holthusen AE, McKeithen TM, McFadden P (2014). The patient perspective on overactive bladder: a mixed-methods needs assessment. BMC Fam Pract..

[114] Palmer MH, Newman DK (2006). Bladder control educational needs of older adults. J Gerontol Nurs..

[115] Teunissen D, van Weel C, Lagro-Janssen T (2005). Urinary incontinence in older people living in the community: examining help-seeking behaviour. Br J General Pract..

[116] Andersson G, Johansson J-E, Nilsson K, Sahlberg-Blom E (2008). Accepting and adjusting: older women's experiences of living with urinary incontinence. Urol Nurs..

[117] Andersson G, Johansson JE, Nilsson K, Sahlberg-Blom E (2009). Perceptions of urinary incontinence among Syrian Christian women living in Sweden. J Transcult Nurs..

[118] Ashworth PD, Hagan MT (1993). The meaning of incontinence: a qualitative study of non-geriatric urinary incontinence sufferers. J Adv Nurs..

[119] Cochran A (1998). Response to urinary incontinence by older persons living in the community. J Wound Ostomy Continence Nurs..

[120] Doshani A, Pitchforth E, Mayne CJ, Tincello DG (2007). Culturally sensitive continence care: a qualitative study among south Asian Indian women in Leicester. Fam Pract..

[121] Dowd TT (1991). Discovering older women's experience of urinary incontinence. Res Nurs Health..

[122] Getliffe K, Fader M, Cottenden A, Jamieson K, Green N (2007). Absorbent products for incontinence: 'treatment effects' and impact on quality of life. J Clin Nurs..

[123] Gjerde JL, Rortveit G, Muleta M, Blystad A (2013). Silently waiting to heal: experiences among women living with urinary incontinence in Northwest Ethiopia. Int Urogynecol J..

[124] Griffiths F, Pepper J, Jørstad-Stein EC, Smith JF, Hill L, Lamb SSE (2009). Group versus individual sessions delivered by a physiotherapist for female urinary incontinence: an interview study with women attending group sessions nested within a randomised controlled trial. BMC Womens Health..

[125] Hägglund D, Wadensten B (2007). Fear of humiliation inhibits women's care-seeking behaviour for long-term urinary incontinence. Scand J Caring Sci..

[126] Hägglund D, Ahlström G (2007). The meaning of women's experience of living with long-term urinary incontinence is powerlessness. J Clin Nurs..

[127] Hamid TA, Pakgohar M, Ibrahim R, Dastjerdi MV (2015). ‘Stain in life’: the meaning of urinary incontinence in the context of Muslim postmenopausal women through hermeneutic phenomenology. Arch Gerontol Geriatr..

[128] Hayder D, Schnepp W (2010). Experiencing and managing urinary incontinence: a qualitative study. West J Nurs Res..

[129] Hayder D (2012). The effects of urinary incontinence on sexuality: seeking an intimate partnership. J Wound Ostomy Continence Nurs..

[130] Higa R, Chvatal VLS, de Moraes Lopes MHB, Turato ER (2011). The meanings of silence in Brazilian women with urinary incontinence. J Wound Ostomy Continence Nurs..

[131] Horrocks S, Somerset M, Stoddart H, Peters TJ (2004). What prevents older people from seeking treatment for urinary incontinence? A qualitative exploration of barriers to the use of community continence services. Fam Pract..

[132] Jackson CB, Botelho EM, Welch LC, Joseph J, Tennstedt SL (2012). Talking with others about stigmatized health conditions: implications for managing symptoms. Qual Health Res..

[133] Kao H-T, Hayter M, Hinchliff S, Tsai C-H, Hsu M-T (2015). Experience of pelvic floor muscle exercises among women in Taiwan: a qualitative study of improvement in urinary incontinence and sexuality. J Clin Nurs..

[134] Komorowski L, Chen B (2006). Female urinary incontinence in China: experiences and perspectives. Health Care Women Int..

[135] Li FLW, Low LPL, Lee DTF (2007). Chinese women's experiences in coping with urinary incontinence. J Clin Nurs..

[136] MacInnes CL (2008). Why women leave therapy for stress incontinence. Nurs Times..

[137] Mason L, Glenn S, Walton I, Appleton C (1999). The experience of stress incontinence after childbirth. Birth (Berkeley, Calif).

[138] Mason L, Glenn S, Walton I, Hughes C (2001). Women’s reluctance to seek help for stress incontinence during pregnancy and following childbirth. Midwifery..

[139] Milne JL, Moore KN (2006). Factors impacting self-care for urinary incontinence. Urol Nurs..

[140] Nicolson P, Kopp Z, Chapple CR, Kelleher C (2008). It's just the worry about not being able to control it! A qualitative study of living with overactive bladder. Br J Health Psychol..

[141] Peake S, Manderson L (2003). The constraints of a normal life: the management of urinary incontinence by middle aged women. Women Health..

[142] Peake S, Manderson L, Potts H (1999). ‘Part and parcel of being a woman’: female urinary incontinence and constructions of control. Med Anthropol Q..

[143] Roos A-M, Thakar R, Sultan AH, Burger CW, Paulus ATG (2014). Pelvic floor dysfunction: women's sexual concerns unraveled. J Sex Med..

[144] Sange C, Thomas L, Lyons C, Hill S (2008). Urinary incontinence in Muslim women. Nurs Times..

[145] Shaw C, Tansey R, Jackson C, Hyde C, Allan R (2001). Barriers to help seeking in people with urinary symptoms. Fam Pract..

[146] Shaw C, Brittain K, Tansey R, Williams K (2008). How people decide to seek health care: a qualitative study. Int J Nurs Stud..

[147] Shaw C, Williams KS, Assassa RP (2000). Patients' views of a new nurse-led continence service. J Clin Nurs..

[148] Siu JY-M (2014). ‘Seeing a doctor is just like having a date’: a qualitative study on doctor shopping among overactive bladder patients in Hong Kong. BMC Fam Pract..

[149] Siu JY-M (2015). Communicating under medical patriarchy: gendered doctor-patient communication between female patients with overactive bladder and male urologists in Hong Kong. BMC Womens Health..

[150] Skoner MM, Haylor MJ (1993). Managing incontinence: women's normalizing strategies. Health Care Women Int..

[151] St John W, James H, McKenzie S (2002). ‘Oh, that's a bit of a nuisance’: community-dwelling clients ' perspectives of urinary continence health service provision. J Wound Ostomy Continence Nurs..

[152] van den Muijsenbergh ME, Lagro-Janssen TA (2006). Urinary incontinence in Moroccan and Turkish women: a qualitative study on impact and preferences for treatment. Br J General Pract..

[153] Welch LC, Botelho EM, Joseph JJ, Tennstedt SL (2012). A qualitative inquiry of patient-reported outcomes: the case of lower urinary tract symptoms. Nurs Res..

[154] Welch LC, Taubenberger S, Tennstedt SL (2011). Patients' experiences of seeking health care for lower urinary tract symptoms. Res Nurs Health..

[155] Wilkinson K (2001). Pakistani women's perceptions and experiences of incontinence. Nurs Standard (Royal College of Nursing (Great Britain) : 1987).

[156] Zeznock DE, Gilje FL, Bradway C (2009). Living with urinary incontinence: experiences of women from 'The last frontier'. Urol Nurs..

[157] Hatchett L, Hebert-Beirne J, Tenfelde S, Lavender MD, Brubaker L (2011). Knowledge and perceptions of pelvic floor disorders among african american and Latina women. Female Pelvic Med Reconstr Surg..

[158] Welch LC, Botelho EM, Tennstedt SL (2011). Race and ethnic differences in health beliefs about lower urinary tract symptoms. Nurs Res..

[159] Wells M, Wagg A (2007). Integrated continence services and the female Bangladeshi population. Br J Nurs..

[160] Akyuz A, Kok G, Kilic A, Guvenc G (2014). In her own words: living with urinary incontinence in sexual life. Sex Disabil..

[161] Coyne KS, Margolis MK, Jumadilova Z, Bavendam T, Mueller E, Rogers R (2007). Overactive bladder and women's sexual health: what is the impact?. J Sex Med..

[162] Delarmelindo RCA, CMGdL P, RAP R, SCM B (2013). Between suffering and hope: rehabilitation from urinary incontinence as an intervening component. Ciênc Saúde Colet..

[163] Siu L-SK, Lopez V (2012). Chinese women's experiences of stress incontinence: a descriptive qualitative study. Int J Urol Nurs..

[164] Elstad EA, Taubenberger SP, Botelho EM, Tennstedt SL (2010). Beyond incontinence: the stigma of other urinary symptoms. J Adv Nurs..

[165] Brown JS, Subak LL, Gras J, Brown BA, Kuppermann M, Posner SF (1998). Urge incontinence: The patient's perspective. J Womens Health.

[166] Klemm LW, Creason NS (1991). Self-care practices of women with urinary incontinence--a preliminary study. Health Care Women Int.

[167] Higa R, CRSFd R, Campos LK, MHdM L, Turato ER (2010). Vivências de mulheres brasileiras com incontinência urinária. Texto Contexto Enfermagem..

[168] Siddiqui NY, Ammarell N, Wu JM, Sandoval JS, Bosworth HB (2016). Urinary incontinence and health-seeking behavior among White, black, and Latina women. Female Pelvic Med Reconstr Surg..

[169] Vethanayagam N, Orrell A, Dahlberg L, McKee KJ, Orme S, Parker SG, Gilhooly M (2017). Understanding help-seeking in older people with urinary incontinence: an interview study. Health Soc Care Community..

[170] Douglas M (1966). Purity and danger: an analysis of the concepts of pollution and taboo.

[171] Turner V (1966). The ritual process structure and anti-structure.

[172] Talcott-Parsons (1951). The social system.

[173] National-Institute-for-Health-and-Care-Excellence: Urinary incontinence in women: management - Clinical Guideline. 2013. https://www.niceorguk/guidance/cg171/resources/urinary-incontinence-in-women-management-pdf-35109747194821.

[174] Hay-Smith EJC, Ryan K, Dean S (2007). The silent, private exercise: experiences of pelvic floor muscle training in a sample of women with stress urinary incontinence. Physiotherapy..

[175] Hemachandra NN, Rajapaksa LC, Manderson L (2009). A ‘usual occurrence:’ stress incontinence among reproductive aged women in Sri Lanka. Soc Sci Med..

[176] Kincade JE, Johnson TM, Ashford-Works C, Clarke MK, Busby-Whitehead J (1999). A pilot study to determine reasons for patient withdrawal from a pelvic muscle rehabilitation program for urinary incontinence. J Appl Gerontol..

[177] Teunissen D, Van Den Bosch W, Van Weel C, Lagro-Janssen T (2006). ‘It can always happen’: the impact of urinary incontinence on elderly men and women. Scand J Prim Health Care..

[178] Hamid T-A, Ibrahim R, Vahid-Dastjerdi M (2015). Portrait representation of postmenopausal Women’s experiences of living with urinary incontinence AU - Pakgohar. Minoo. J Women Aging..

[179] Roin A, Nord C (2015). Urine incontinence in women aged sixty to sixty-five: negotiating meaning and responsibility. Scand J Caring Sci..

